# Chemogenetic E-MAP in *Saccharomyces cerevisiae* for Identification of Membrane Transporters Operating Lipid Flip Flop

**DOI:** 10.1371/journal.pgen.1006160

**Published:** 2016-07-27

**Authors:** Hector M. Vazquez, Christine Vionnet, Carole Roubaty, Shamroop k. Mallela, Roger Schneiter, Andreas Conzelmann

**Affiliations:** Division of Biochemistry, Department of Biology, University of Fribourg, Fribourg, Switzerland; Stanford University School of Medicine, UNITED STATES

## Abstract

While most yeast enzymes for the biosynthesis of glycerophospholipids, sphingolipids and ergosterol are known, genes for several postulated transporters allowing the flopping of biosynthetic intermediates and newly made lipids from the cytosolic to the lumenal side of the membrane are still not identified. An E-MAP measuring the growth of 142'108 double mutants generated by systematically crossing 543 hypomorphic or deletion alleles in genes encoding multispan membrane proteins, both on media with or without an inhibitor of fatty acid synthesis, was generated. Flc proteins, represented by 4 homologous genes encoding presumed FAD or calcium transporters of the ER, have a severe depression of sphingolipid biosynthesis and elevated detergent sensitivity of the ER. *FLC1*, *FLC2* and *FLC3* are redundant in granting a common function, which remains essential even when the severe cell wall defect of *flc* mutants is compensated by osmotic support. Biochemical characterization of some other genetic interactions shows that Cst26 is the enzyme mainly responsible for the introduction of saturated very long chain fatty acids into phosphatidylinositol and that the GPI lipid remodelase Cwh43, responsible for introducing ceramides into GPI anchors having a C26:0 fatty acid in *sn-*2 of the glycerol moiety can also use lyso-GPI protein anchors and various base resistant lipids as substrates. Furthermore, we observe that adjacent deletions in several chromosomal regions show strong negative genetic interactions with a single gene on another chromosome suggesting the presence of undeclared suppressor mutations in certain chromosomal regions that need to be identified in order to yield meaningful E-map data.

## Author Summary

All living cells define their boundaries by lipid-containing membranes, which are impermeable to ions and water-soluble metabolic intermediates, and thus allow maintaining constant conditions inside the cells and stopping metabolic intermediates from diffusing away. Membranes are formed by amphiphilic lipids that have a hydrophilic and a hydrophobic component. Such lipids form flat double-layered sheets (bilayers) wherein the hydrophilic components of the constituent lipids are directed towards the aqueous surroundings, the hydrophobic ones populate the center of the bilayer. Membranes grow when enzymes resident in the bilayer synthesize new amphiphilic lipids. These enzymes have their active site on one side of the membrane and insert the newly made lipids in only one of the two layers. To ensure symmetric growth of membranes, cells need flippases catalyzing the transfer of lipids from one into the other layer. To identify unknown flippases we performed a chemogenetic interaction screen able to bring to light functions of unknown proteins through their genetic interaction with genes of known function. The data point to Flc proteins as potential lipid flippases of the endoplasmic reticulum, reveal novel lipid modifying activities of Cst26 and Cwh43 and suggest that undeclared suppressor mutations in certain chromosomal regions can generate false genetic interactions.

## Introduction

All living cells define their boundaries by lipid-containing membranes, which in eukaryotes are mainly made of glycerophospholipids (GPLs), sphingolipids and sterols. Phosphatidic acid (PA) is an obligatory intermediate in the biosynthesis of all GPLs [1,2]. In eukaryotes, PA is generated through acyl-CoA dependent reactions from glycerol-3-phosphate (G3P), which is acylated twice, first on *sn*-1 by a G3P acyltransferase (GPAT) and subsequently on *sn*-2 by a 1-acylglycerol-3-phosphate acyltransferase (AGPAT). PA is then used for the biosynthesis of mature GPLs such as phosphatidylserine (PS), phosphatidylcholine (PC), phosphatidylinositol (PI) and several others. Similarly, mature sphingolipids are built on ceramides, which are synthesized by acyl-CoA dependent ceramide synthases attaching a fatty acid (FA) to the free amino group of sphingoid bases. In eukaryotes most of these biosynthetic reactions occur in the ER, but some are also present in mitochondria and in peroxisomes. For the enzymes required to make PA, sphingolipids and sterols, biochemical assays are available and corresponding genes are identified.

There is strong evidence that in certain bacteria GPATs and AGPATs belonging to the large lysophospholipid acyltransferase superfamily (pfam01553, COG0204 or COG2937) have their active site at the cytosolic side of the cytosolic membrane [3–7]. As for eukaryotes, the traditional view is that all acylation reactions in the biosynthesis of PA and triacylglycerides (TAGs) as well as the further elaboration of GPLs occur on the cytosolic side of membranes (or else, the matrix side of chloroplasts) [1,8,9] but some notable exceptions may exist. Recent data indicate that the active site of some membrane bound O-acyltransferases (MBOATs) probably resides in the ER lumen. According to the Pfam database (http://pfam.xfam.org/) this pfam03062 family of multispan integral membrane proteins presently comprises 16226 sequences belonging to 9933 bacterial and eukaryotic organisms [10]. Many MBOAT proteins acylate lipids such as cholesterol, diacylglycerol (DAG), or lyso-GPLs, but some acylate ER lumenal secretory proteins such as Hedgehog, Wnt, Ghrelin and yeast glycosylphosphatidylinositol (GPI) anchored proteins [11–15]. Likewise biochemical investigations localize the highly conserved putative active site His residue of MBOAT proteins to the ER lumen [10,16–20]. Moreover, the GPI biosynthetic intermediate PI-Glucosamine is acylated on the inositol moiety in the ER lumen and lyso-GPI anchors are acylated in the lumen of the Golgi, reactions achieved by two acyltransferases not belonging to the MBOAT family [21,22]. Acyl-CoA dependent acylation reactions in the lumen of the secretory apparatus imply that acyl-CoA or its acyl group may have to be transported through organellar membranes. Such transport is well documented for the import of fatty acids into mitochondria and peroxisomes, whereby in this latter case it is unclear if acyl-CoA or only acyls are crossing the membrane [23,24]. No acyl-CoA transporters however have been described for the ER or Golgi. Besides, the non-specific GPL flippase of the ER has not yet been identified genetically [6,25] although the genes encoding the flippases for aminophospholipids at the plasma membrane and Golgi and their antagonists, the scramblases of the plasma membrane are well characterized [6,25].

## Results and Discussion

### Generation of a multispan protein E-MAP for detection of lipid flippases

Here we report on a genetic screen aiming at the detection of genes required for the transfer of GPLs, acyl-CoAs, other acyls or GPl intermediates across organellar membranes.

**Fig 1:**
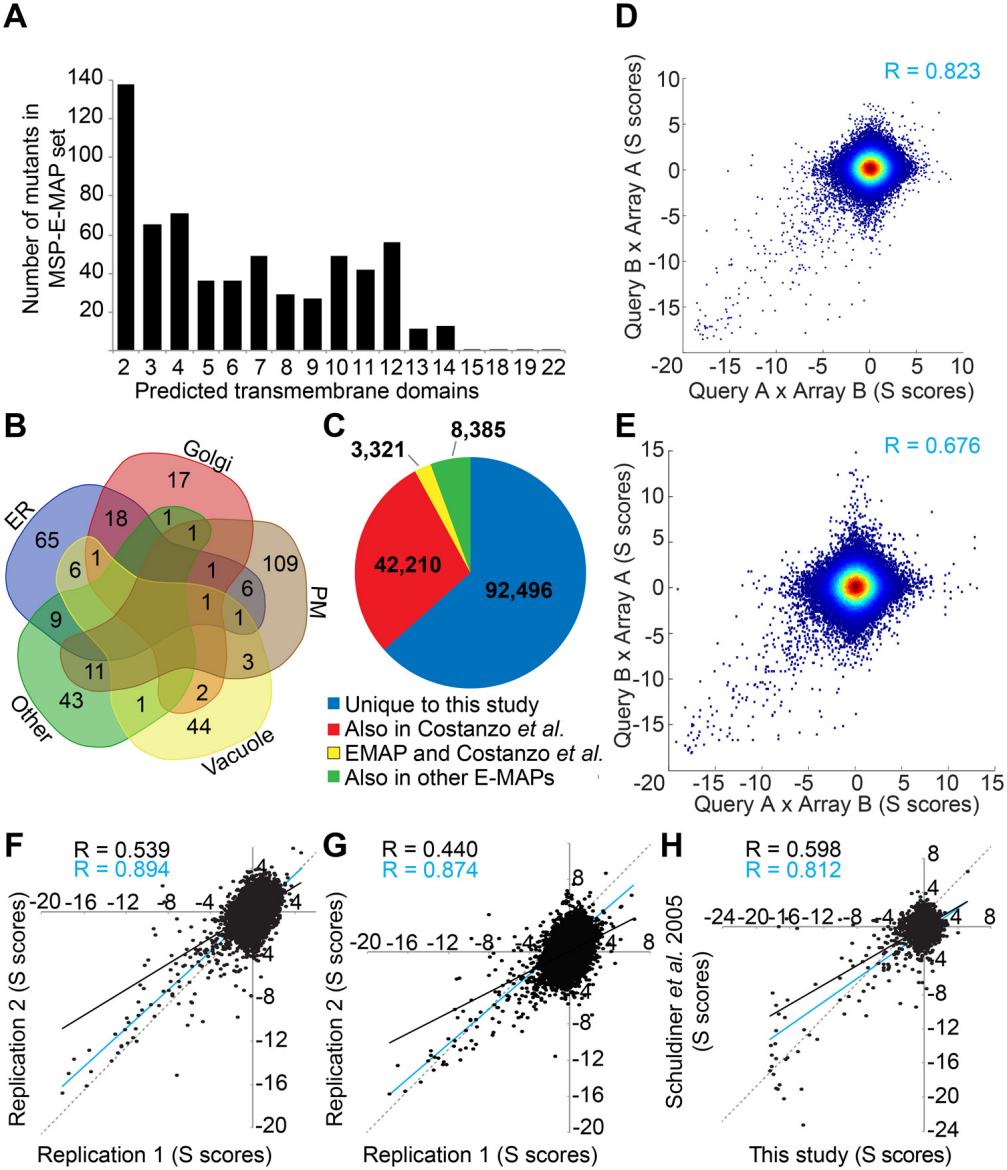
Membrane spanning protein E-MAP. (A) number of predicted TMDs in the proteins of the E-MAP gene set. (B) using manually curated annotations of YeastMine (http://yeastmine.yeastgenome.org/) most genes of the E-MAP set were attributed to one of 4 different subcellular locations (S1 Table). A few genes are described as being in two or three locations. PM = plasma membrane. (C) overlap of gene combinations tested in our MSP-E-MAP, the E-MAPs form the Krogan lab (http://interactome-cmp.ucsf.edu/) and systematic genetic array (SGA) studies of the Boone lab (http://drygin.ccbr.utoronto.ca/). (D, E) comparison of unaveraged genetic interaction scores (S scores) obtained in reciprocal crosses of [query A x array B] and [query B x array A] for all double mutants in the MSP-E-MAP (D) and MSP/C-E-MAP (E) after removal of noisy strains (see materials and methods). Correlation coefficients of significant S scores are indicated in blue. (F, G) a subset of 84 randomly chosen queries were crossed anew with the 629 genes of the array selecting without (F) or with Cerulenin (G). The experiment (= replicate 2) was done by authors Christine Vionnet and Carole Roubaty and the S scores plotted against those obtained in the first E-MAP experiment done by author Hector Vazquez (= replicate 1). Trend-lines and the corresponding Pearson correlation values of all dots (black) or only those having a statistically significant S score (blue). The dotted lines represent the theoretical correlation of R = 1. H, comparison of the S scores of double mutants of our MSP-E-MAP with the corresponding S scores obtained by [28] in their ESP-E-MAP.

**Fig 2:**
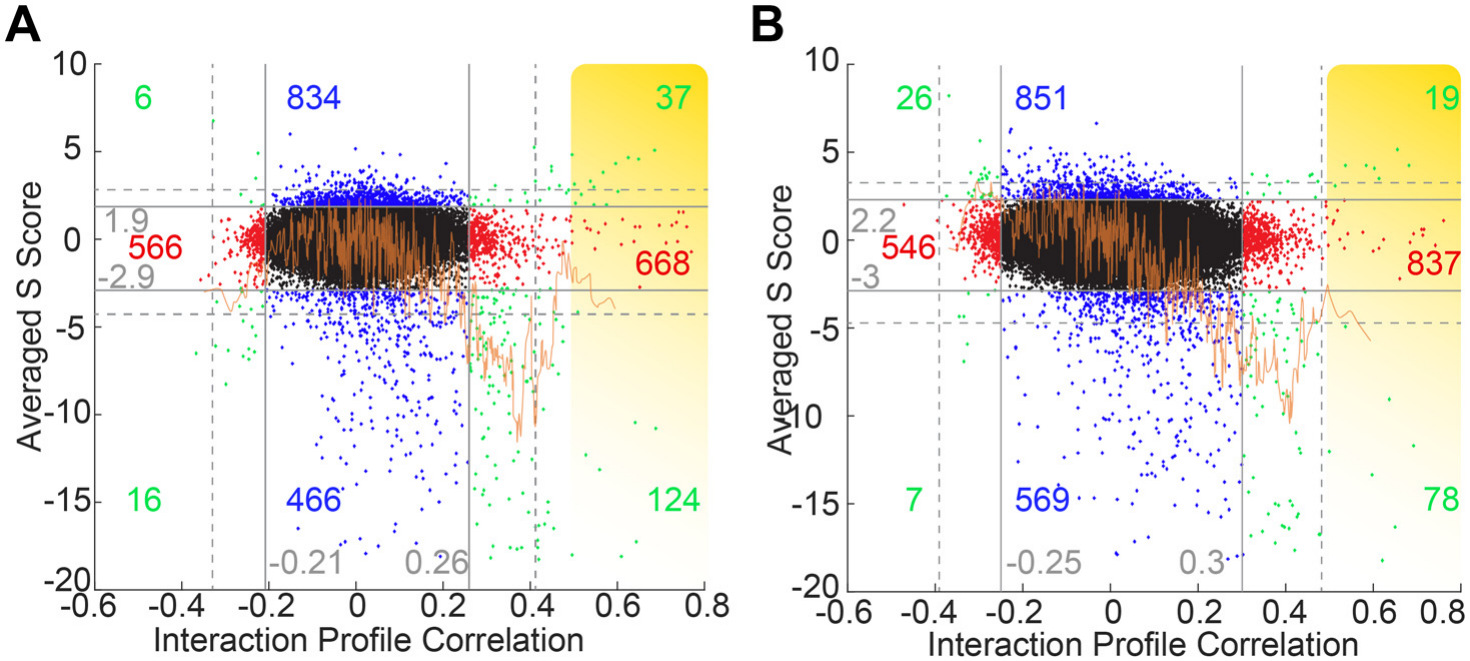
Profile of genetic interactions and correlations. (A, B) S scores and correlations for each of the 147’153 gene pairs of the MSP-E-MAP (A) and the MSP/C-E-MAP done on Cerulenin (B). Each of the 147’153 gene pairs is represented by a dot with its x- and y-coordinates corresponding to the S score and profile correlation, respectively. Horizontal and vertical gray lines separate statistically non-significant from significant values (unadjusted P values ≤ 0.005 for S scores and ≤ 0.05 for correlations, respectively). Significant S scores associated with non-significant correlations are in blue, significant correlations associated with non-significant S scores are in red and significant correlations associated with significant S scores are in green. The yellow zones in (A) and (B) together highlight 68 gene pairs with correlations > 0.5 listed in S2C Table. Dotted lines indicate significance thresholds (P<0.05) after Bonferroni corrections (S2B Table, S3 Text, Materials and Methods). The orange lines indicate the average of significant S scores as a function of the correlation coefficient obtained with a window of 10 values.

We argued that such lipid flipping transporters might be redundant, in part explaining why they have not yet been identified genetically. We also hypothesized that any growth phenotype due to a lack of some lipid flipping activity in a double mutant may be enhanced if the corresponding lipid is made in reduced amounts. We further assumed that, similar to already characterized lipid flipping transporters [26,27] lipid flippases would need to have multiple transmembrane domains (TMDs), i.e. belong to the so called multispan proteins (MSPs). We thus set out to create an E-MAP from a set of 629 strains harboring a deletion or mutation in a MSP with the intent to compare the fitness of double mutants in the absence or presence of Cerulenin, a relatively specific inhibitor of the FA synthase. In the following these E-MAPs are called MSP-E-MAP and MSP/C-E-MAP (C for Cerulenin), respectively. Indeed, all known membrane lipids except for ergosterol contain FAs or derivatives of them. The MSP set contained numerous proteins with 2–14 and in rare cases up to 22 predicted TMDs (Fig 1A). For 340 genes, the gene product had been localized at a particular subcellular location (Fig 1B, S1 Table). Our MSP-E-MAP set was also subdivided manually into 11 different functional categories and was strongly enriched in transporters and lipid biosynthetic enzymes (S1 Table). After data clean up (S3_supplemental material, materials and methods) the MSP- or MSP/C-E-MAPs contained 606 or 654 significant negative, and 877 or 896 significant positive interactions, respectively (unadjusted P value <0.005, see S3_supplemental material, materials and methods)(Fig 2A and 2B; S2B Table).

### Quality of E-MAP data

When comparing (in May 2015) the genetic interactions with those reported by the Krogan and Boone labs, our MSP-E-MAP tested 92’496 genetic interactions not tested before (Fig 1C). E-MAPs require that the 543 genes of the set be deleted once in the *MAT***a** background to generate the array, once in the *MAT***α** background to generate the query set. After removal of noisy strains (see S3 Text, Materials and Methods) the significant S scores obtained in reciprocal [query A x array B] and [query B x array A] crosses where strongly correlated in the MSP-E-MAP (Fig 1D) as well as the MSP/C-E-MAP (Fig 1E). Significant positive interactions were found in both MSP/C- and MSP-E-MAP, but in the former some clustered along x- and y-axes, hence were not found in reciprocal crosses (Fig 1E; S1A–S1D Fig (Processing of raw E-MAP data)). This may be caused by additional noise due to the addition of Cerulenin but we can’t exclude that there is a biological significance to this phenomenon. To ascertain reproducibility, part of the E-MAP was repeated, i.e. 84 randomly chosen queries were once more crossed with all arrays and analyzed both in the presence and absence of Cerulenin. As seen in Fig 1F, 1G and S2 Fig (Reproducibility of correlations of the MSP-E-MAP and MSP/C-E-MAP), the S scores and correlations of the first complete and the second partial E-MAP were highly correlated, and became even much more correlated when only significant values were considered.

As shown in Fig 1H, there also was good agreement between S scores in our MSP-E-MAP with the corresponding interactions in the **e**arly **s**ecretory **p**athway E-MAP (ESP-E-MAP) [28,29], which inaugurated the E-MAP approach. In an E-MAP, the genetic interactions of a given mutant with all other mutants generates a characteristic interaction profile and any two mutants get a correlation score describing the similarity of their interaction profiles, whereby a very positive correlation of interaction profiles suggests collaboration of the two genes for a single function [28]. As in the ESP-E-MAP report [28], in our MSP-E and MSP/C-MAPs the average of S scores as a function of the correlation score gets increasingly negative, up to a correlation value of about +0.4, and abruptly becomes more positive at higher correlation values (Fig 2A and 2B). Similarly, the ratio of the number of interactions with significantly positive S scores over the number of interactions with significantly negative S scores drops up to a correlation of about 0.4 and then goes up again at higher correlation values (S1G and S1H Fig (Processing of raw E-MAP data)).

As typical for E-MAPs, our data showed clustering of functionally related genes upon hierarchical clustering and confirmed some genes as "hyper-interactors" since their deletion generated many more interactions than the average deletion. Data also showed preferential interactions between genes of certain functional classes. This and a general statistical analysis of our data are to be found in S1 Text and S8 Fig (Heat maps and main clusters of the MSP-E-MAP), S9 Fig (Enlargement of regions in heat maps of S8A and S8B Fig showing frequent interactions or correlations between genes belonging to two different clusters), S10 Fig (Frequency of significant interactions and correlations within and amongst different functional classes of genes) and S11 Fig (Interdependence of the number of interactions and correlations generated by the MSP-E-MAP).

### Flc proteins are implicated in an essential process

There were significant changes between the MSP- and the MSP/C-E-MAP in the sense that some genetic interactions were aggravated, others alleviated on Cerulenin as described in S2 Text and S12 Fig (Comparison of E-MAPs with or without Cerulenin). We had hoped that potential lipid transporters could surface as pairs of less well characterized genes, which would interact more negatively on Cerulenin than without. However, we could not find many such pairs in our data. Most candidate genes were unattractive because of their known localization in the mitochondria, the vacuole or at the plasma membrane, because their interactions were with well-characterized genes not involved in lipid biosynthesis, or because they failed to generate strongly negative S scores.

**Fig 3:**
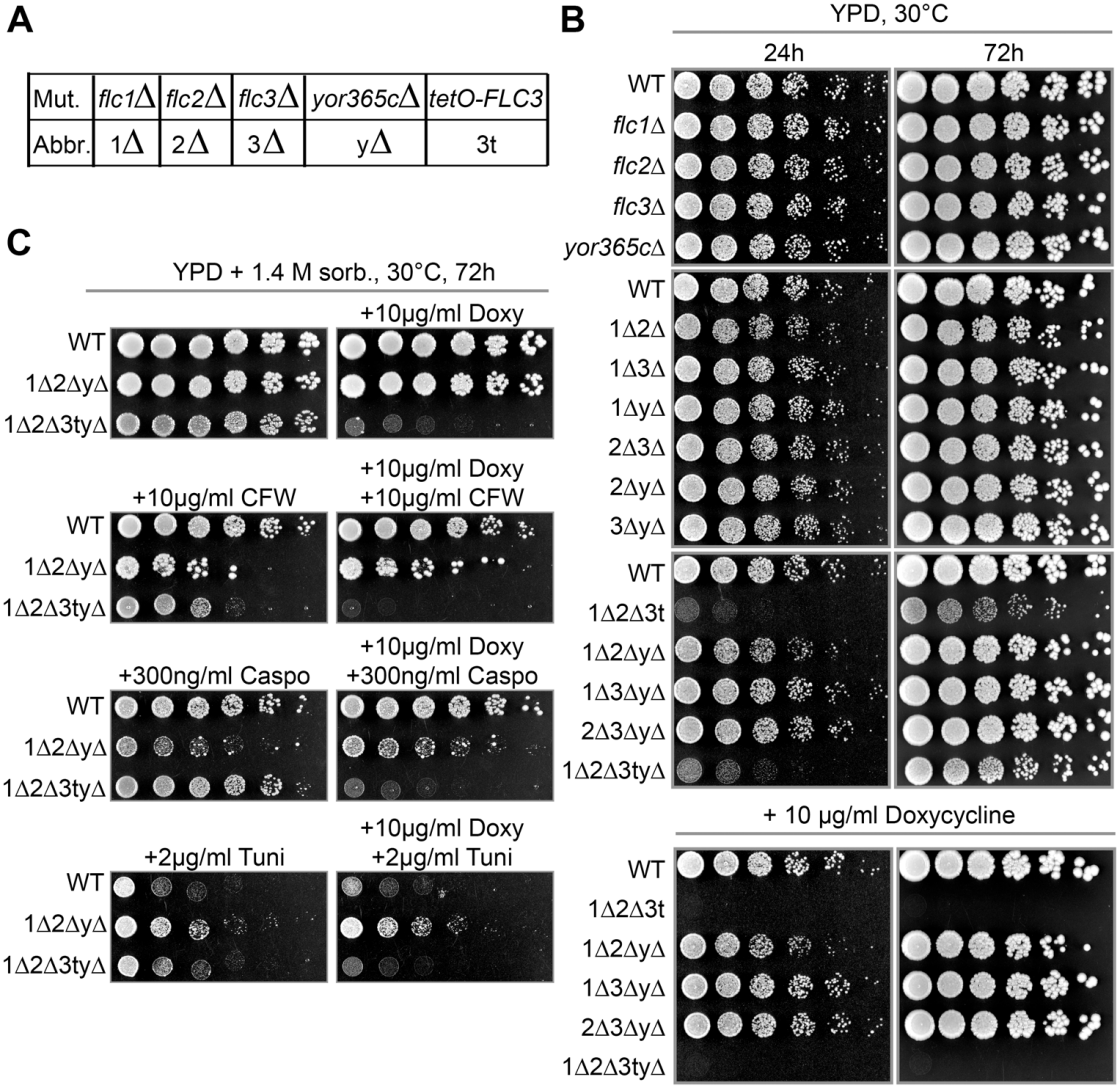
Growth and drug resistance of *flc* mutants. (A) abbreviations used in figures and text for double, triple and quadruple mutants in *flc* genes. (B, C) four fold serial dilutions of WT, single or combined *flc* mutants were spotted on YPD (B), or YPD + 1.4 M sorbitol (C), with or without different cell wall destabilizing drugs and/or Doxy to downregulate transcription of tetO-*FLC3*.

Therefore, we turned our attention to gene pairs giving strong negative interactions in both the MSP- and the MSP/C-E-MAP, which were not strongly aggravated on Cerulenin and in which the function is not fully understood. These criteria are met by the Flc proteins: The S score of the *flc1*Δ *flc2*Δ double mutant on Cerulenin drops from *-11*.*8* to *-12*.*7*, but the double mutant contains two further paralogs, *FLC3* and *YOR365c*, which still are in their wild type (WT) state. Flc proteins are widely conserved in fungi, and have three domains: 1) an N-terminal hydrophilic domain of 150–200 amino acids forming a lipid binding pocket (also present in the human Niemann-Pick type C2 protein required for the egress of cholesterol from late endosomes), 2) a 450 amino acids long very hydrophobic region with >8 predicted TMDs, which is classified as a TRP (transient receptor potential, pfam 06011) domain and is related to human mucolipin and polycystin2 calcium transporters, and finally 3) a hydrophilic, 100 to 200 amino acids long non-conserved C-terminus. Flc1, Flc2 and Flc3 have been localized in the ER and Golgi, whereas Yor365c was reported to be mitochondrial [30,31]. In a previous report the *flc1*Δ *flc2*Δ strain was found to be nonviable, but growth was partially rescued by 1 M sorbitol, was hypersensitive to the chitin-binding drug calcofluor white (CFW), and cells had thickened cell walls, diminished N-glycan elongation in the Golgi, reduced β1,6 glucan synthesis at the plasma membrane, a delay in the maturation of the vacuolar protease CPY and a reduced FAD import into the ER of permeabilized spheroplasts [30]. Furthermore, a recent study proposes that Flc1, Flc2 and Flc3 mediate or regulate calcium release from intracellular stores into the cytosol in response to hypotonic shock [31]. As shown in Fig 3B and S3 Fig (Division times of single or combined *flc* mutants), in our background *flc1*Δ *flc2*Δ, *flc1*Δ *flc3*Δ and *flc2*Δ *flc3*Δ double mutant strains were all viable, even if *YOR365c* was deleted in addition. *Flc1*Δ *flc2*Δ had a reduced growth that confirmed the negative genetic interaction seen in our E-MAP. However, the *flc1*Δ *flc2*Δ *flc3*Δ triple mutant was not viable and this argues that the three Flc proteins are redundant with regard to an essential function. We thus created *flc1*Δ *flc2*Δ *tet*_*off*_*flc3* strains, in which the tetracycline repressible tet_off_ promoter replaced the genomic *FLC3* promoter. These mutants showed slower growth than *flc1*Δ *flc2*Δ cells and ceased to grow on Doxy (Fig 3B and S3 Fig (Division times of single or combined *flc* mutants)). Further deletion of *YOR365c* had no negative influence on the growth of *flc1*Δ *flc2*Δ *tet*_*off*_*flc3* cells. To resolve whether this essential function is restricted to the maintenance of cell wall integrity (CWI), we placed *flc1*Δ *flc2*Δ *tet*_*off*_*flc3 yor365c*Δ (in the following abbreviated as 1Δ2Δ3tyΔ) on 1.4 M sorbitol. Sorbitol greatly increased viability of 1Δ2Δ3tyΔ but could not rescue cells on Doxy (Fig 3C vs. 3B), arguing that the essential function of Flc proteins involves more than granting osmoresistance, one of the major tasks of the cell wall. In comparison to WT, 1Δ2Δ3tyΔ were much more sensitive to CFW, as well as to the β1,3glucan synthase inhibitor caspofungin, and the inhibitor of N-glycosylation tunicamycin, but, when placed on sorbitol, they remained only sensitive to CFW (Fig 3C). This argues that sorbitol is efficiently combating the cell wall deficiency of 1Δ2Δ3tyΔ, but does not eliminate it completely since CFW hypersensitivity signals an elevated chitin content of the cell wall, suggesting that chitin synthesis remains elevated since the cells continue to sense a cell wall problem [32–34].

### Analysis of lipid biosynthesis in *flc* mutants

In an attempt to get evidence for a hypothetical lipid flippase activity of Flc proteins we used standard tests to measure lipid biosynthesis in *flc* mutants.

**Fig 4:**
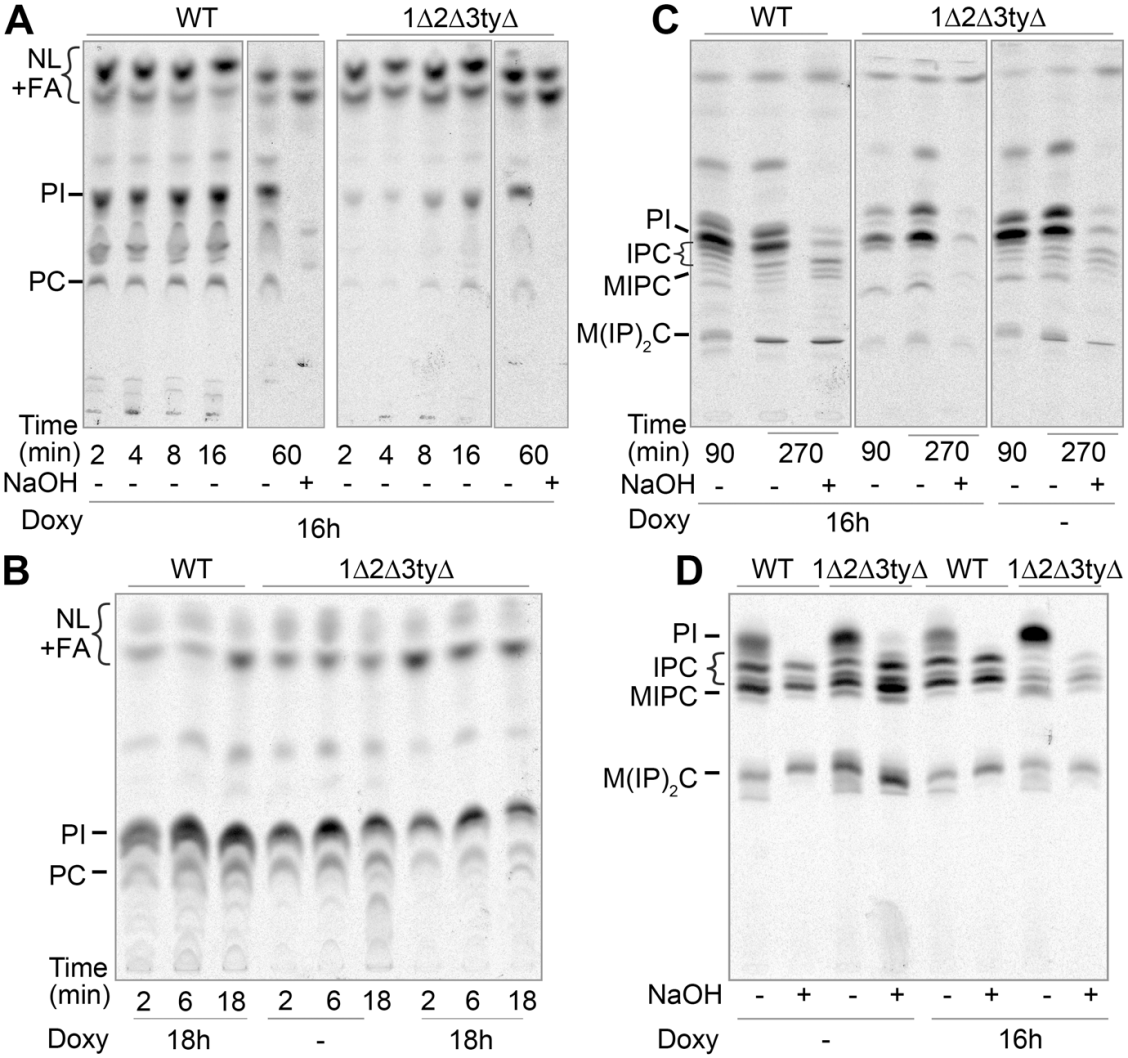
Lipid biosynthesis of 1Δ2Δ3tyΔ mutants assessed by metabolic labeling. (A–C) WT or 1Δ2Δ3tyΔ cells incubated with or without 10 μg/ml Doxy for the indicated times in YPD (A) or YPD + 1.4 M sorbitol (B), were radiolabeled with [^3^H]-C16:0 for the indicated times at 30°C, the lipids were extracted, treated with NaOH as indicated, spotted and separated by TLC using solvent 1. (C) same as in (B) but additional 25 μM of cold C16:0 were added during labeling and lipids were separated by TLC with solvent 2. (D) cells grown with or without 10 μg/ml Doxy for 16 h in inositol-free SC + 1.4 M sorbitol were labeled with [^3^H]-*myo*-inositol for 2 h and the lipids from 10 OD_600_ of each cell type were processed as in (C). NL = neutral lipids are free FAs, DAG, TAG, ergosterol esters and (acyl-)ceramides.

For this 1Δ2Δ3tyΔ cells were labeled with [^3^H]-C16:0 or [^3^H]-*myo*-inositol after 16 h of culture with or without Doxy, the time it takes to see a slow down of the growth rate of Doxy-treated cells in comparison with non-treated cells (S3A Fig (Division times of single or combined *flc* mutants)). When the cells were grown with Doxy in the absence of 1.4 M sorbitol, [^3^H]-C16:0 incorporation into GPLs and sphingolipids in 1Δ2Δ3tyΔ cells was very low (Fig 4A). (Sphingolipids are the only polar lipids remaining after NaOH treatment). When grown in sorbitol, the synthesis rate of GPLs was brought back, although not to WT levels (Fig 4B). This difference could not be attributed to a difference in cell viability since colony forming units (CFU) after 16 h of culture on Doxy with and without sorbitol was the same (39 and 41%, respectively, compared to cells not treated with Doxy). In spite of reduced viability by this criterion, all cells still retained a full redox potential (see below). Differently from GPLs, sphingolipid biosynthesis remained inefficient in Doxy treated 1Δ2Δ3tyΔ cells even on sorbitol, both if [^3^H]-C16:0 or [^3^H]-*myo*-inositol was used to label cells (Fig 4C and 4D). In Doxy treated 1Δ2Δ3tyΔ mutants, the incorporation of [^3^H]-*myo*-inositol tended to remain in the form of phosphatidylinositol (PI) (Fig 4D). Accumulation of PI is expected when ceramides are not made in sufficient quantity since most PI is normally consumed by Aur1-Kei1 transferring inositol-phosphate from PI to ceramides thus generating inositolphosphorylceramides.

Three possible reasons for the reduction of the lipid biosynthesis rate in Doxy treated 1Δ2Δ3tyΔ mutants came to our mind: 1) The proposed lack of FAD [30] would lead to a severe dysfunction of Ero1 and Pdi1 and a lack of proper oxidative folding of some ER proteins, also affecting enzymes involved in lipid biosynthesis [35]. 2) The reduced activity of the lipid biosynthetic enzymes would be related to the growth arrest the cells undergo when incubated with Doxy and 3) The *flc* mutants would indeed have a defect in flipping acyl-CoA or GPLs from the cytosol into the lumen of the ER.

**Fig 5:**
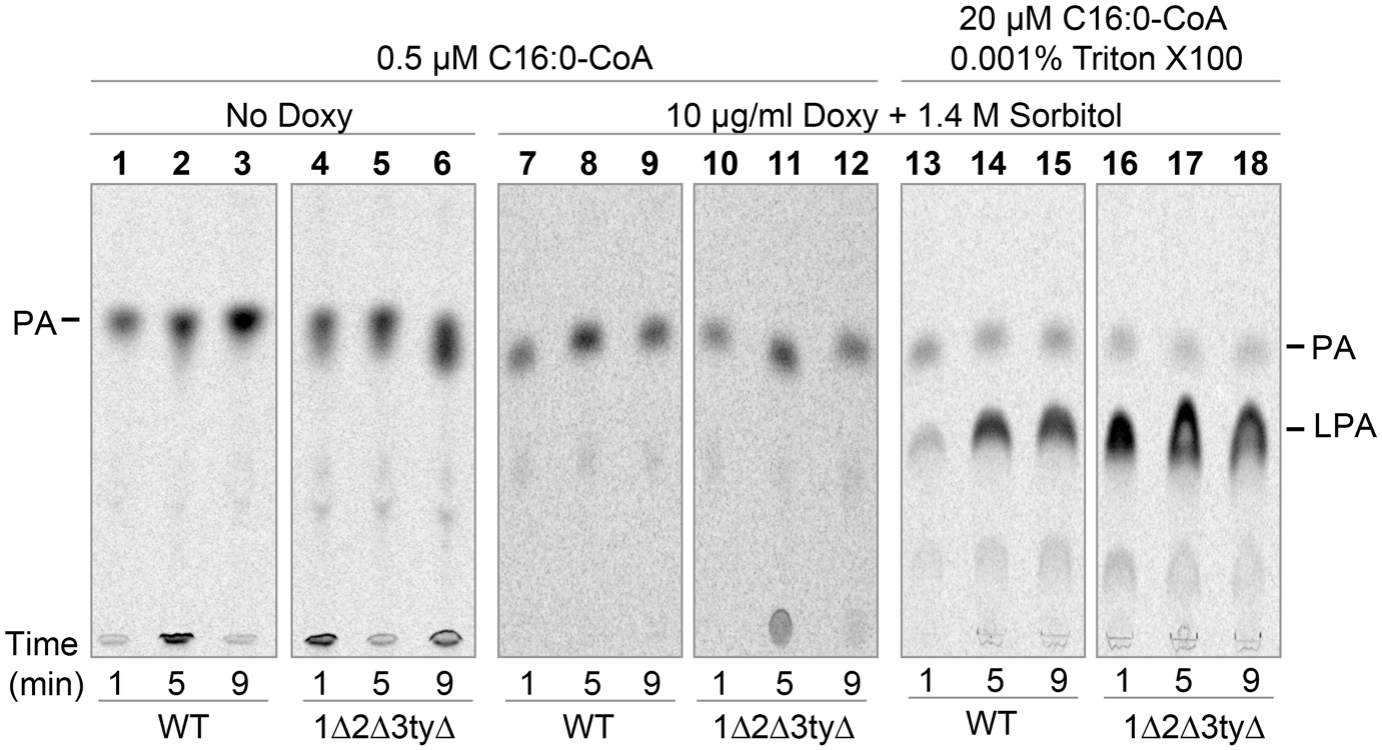
Increased microsomal GPAT and AGPAT activity in *flc* mutants grown on Doxy. Microsomes were produced from WT or 1Δ2Δ3tyΔ cells grown for 16 h in the absence or presence of the indicated concentrations of Doxy and sorbitol, and labeled using 10 μM [^14^C]-G3P (0.5 μCi) and the indicated concentrations of C16:0-CoA and Triton X-100. Lipids were extracted and separated by TLC using solvent 1.

To get evidence for a flippase defect in *flc* mutants, we measured the acyl transferase activity of microsomes in presence of very low concentrations of 16:0-CoA hoping that low concentrations of acyl-CoA would make the PA synthesis more dependent on flippase activities. When microsomes from 1Δ2Δ3tyΔ cells grown on Doxy were incubated with low concentrations of 16:0-CoA (0.5 μM) and a 20 fold excess of [^14^C]-G3P, they made [^14^C]-PA at a normal rate (Fig 5, lanes 1–12). This suggested that Flc proteins were not required for PA biosynthesis and that the ER of 1Δ2Δ3tyΔ cells contained normal GPAT and AGPAT activities. Yet, when the same microsomes were assayed under different conditions, with a 2 fold excess of 16:0-CoA over [^14^C]-G3P plus 0.001% (19 mol %) detergent to permeabilize the microsomes, total incorporation of [^14^C]-G3P into lipids increased as expected, and it appeared that 1Δ2Δ3tyΔ cells grown on Doxy had rather higher GPAT activity than WT (Fig 5, lanes 13–18). This was especially apparent after 1 min of incubation. The normal or elevated microsomal GPAT and AGPAT activities seemed to speak against the possibilities 1 and 2 described above. The increased GPAT activity in microsomes from 1Δ2Δ3tyΔ cells was not easy to interpret: *GPT2* and *SCT1*, encoding the only known GPATs of yeast are not induced during an unfolded protein response [36] but there may be other reasons for enzyme induction or there may be a difference in microsomal membrane lipids allowing detergent to more easily permeabilize the bilayer or to more easily remove some inhibitory component from the GPATs Gpt2 and/or Sct1.

**Fig 6:**
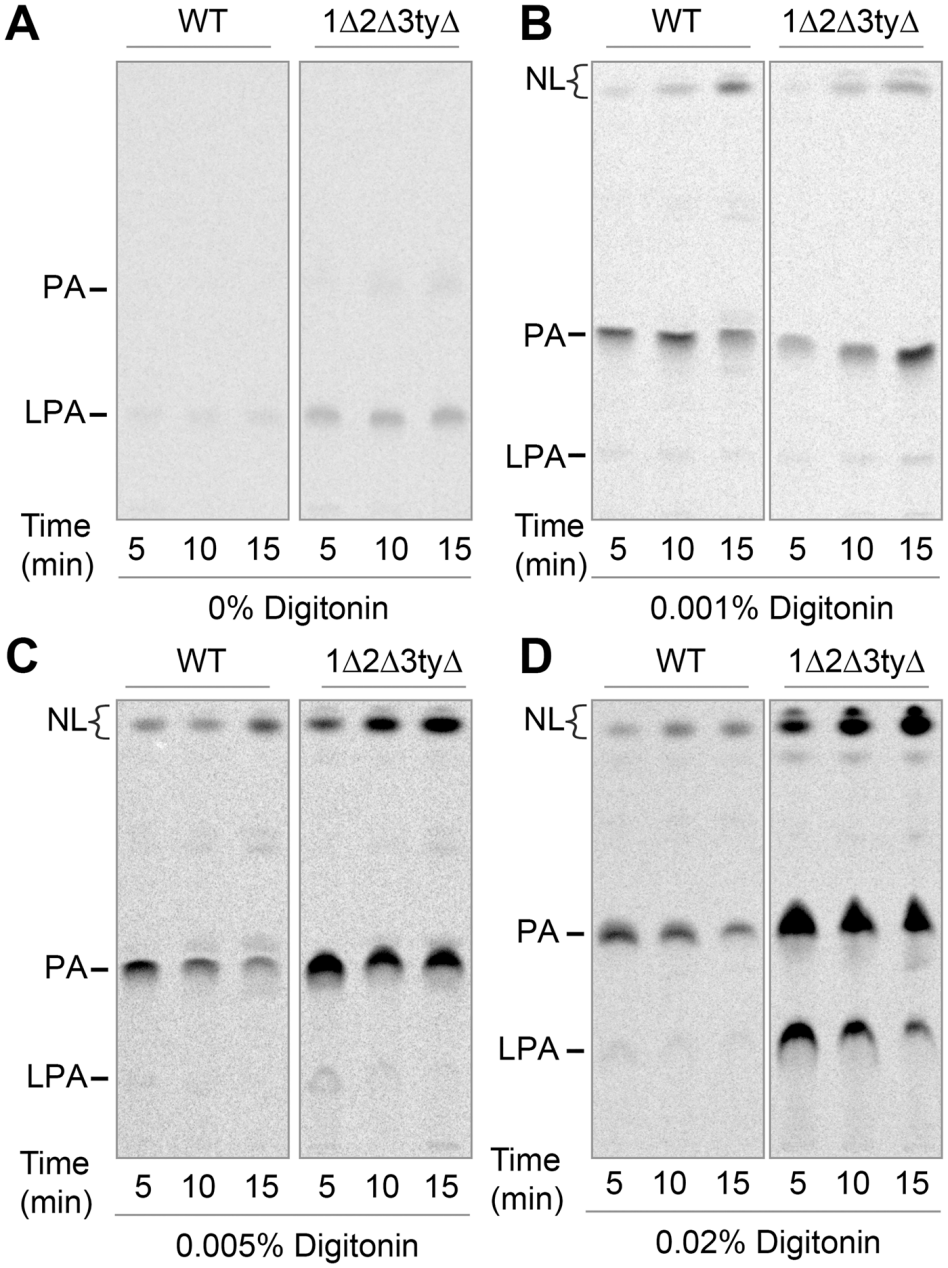
1Δ2Δ3tyΔ mutants have unstable membranes and increased GPAT and AGPAT activities. (A–D) WT or 1Δ2Δ3tyΔ cells grown during 16 h with Doxy in 1.4 M sorbitol, permeabilized for 30 min with indicated concentrations of Digitonin were further incubated with 10 μM C16:0-CoA, 10 μM [^14^C]-G3P (0.5 μCi) for 5 to 15 min. Lipids were extracted and separated by TLC on solvent 1.

Trying to get more evidence for hypothesis 3, i.e. the lack of a lipid flippase in *flc* mutants, we were faced with the dilemma that such lack is anticipated to destabilize the membrane, which is the obligatory support for any biochemical demonstration of flippase activity. It seemed to us that the discrepancy between experiments labeling intact cells and microsomes (Fig 4A and 4B vs. Fig 5) could indicate that 1Δ2Δ3tyΔ cells, because of a flippase defect, produced leaky or even inverted microsomes giving better access of substrates to the active sites of acyltransferases. Therefore, to further explore whether these cells may have a problem with flipping of acyl-CoA or lyso-PA, we opted for the use of intact cells treated with Digitonin to selectively perforate plasma membranes but leaving the ER intact according to a well established method [37,38]. To find the lowest concentrations of Digitonin allowing to permeabilize plasma membranes we used the membrane impermeable 5,5’-dithiobis-2-nitrobenzoic acid (DTNB, Elman’s reagent), which produces a colored compound upon reaction with thiol groups present on cytosolic proteins and glutathione [39]. In this way it was found that the speed of the Elman reaction was dependent on the concentration of Digitonin added in the range of 0 to 0.02% (S4 Fig (Permeabilization of cells with Digitonin and detection of cytosolic thiol groups with DTNB)). It should be noted that in this test the Elman's reagent is in large excess over thiols, so that the reaction reaches plateau when all thiols are oxidized. As these plateaus are the same for WT and 1Δ2Δ3tyΔ cells (S4 Fig (Permeabilization of cells with Digitonin and detection of cytosolic thiol groups with DTNB)), it appears that although 60% of Doxy-treated 1Δ2Δ3tyΔ cells are unable to resume growth and form a colony, i.e. have ceased to be a CFU, they apparently still maintain a normal redox potential and in this sense are alive. S4 Fig (Permeabilization of cells with Digitonin and detection of cytosolic thiol groups with DTNB) shows that in all cell types tested 0.005% Digitonin achieved full, but 0.001% only partial reduction of the reagent within 20 min (see the more detailed description of results in S4 Fig). We therefore hoped that substrates for acyltransferases added to minimally permeabilized cells would enter the cytosol and be used for lipid biosynthesis in a still preserved ER membrane without skirting the potential need for physiological ER based lipid flippases in this process. Thus, WT and 1Δ2Δ3tyΔ cells grown in sorbitol and Doxy were preincubated for 30 min with increasing concentrations of Digitonin as above (S4 Fig (Permeabilization of cells with Digitonin and detection of cytosolic thiol groups with DTNB)) whereupon 10 μM C16:0-CoA plus 10 μM [^14^C]-G3P (0.5 μCi) were added (Fig 6). When no detergent was added, small amounts of [^14^C]-G3P seemed to enter cells and produce mainly lyso-phosphatidic acid (LPA), whereby this phenomenon was more pronounced with 1Δ2Δ3tyΔ than WT cells (Fig 6A). With 0.001% Digitonin, cells started to make significant amounts of phosphatidic acid (PA), which were similar for WT and 1Δ2Δ3tyΔ cells. With 0.005% (17 mol%) Digitonin, 1Δ2Δ3tyΔ cells made significantly more PA and neutral lipids than WT (Fig 6C). PA seemed to be chased with time into DAG or TAG, as if PA synthesis was coming to an early plateau and even early halt. Interestingly, in 1Δ2Δ3tyΔ but not WT cells, the further increase of Digitonin to 0.02% (44 mol%) led to a further increase of activity (Fig 6D), although already 0.005% fully permeabilizes the plasma membrane of all cell types (S4A and S4B Fig (Permeabilization of cells with Digitonin and detection of cytosolic thiol groups with DTNB)). This suggests a basic difference between 1Δ2Δ3tyΔ and WT cells in that in the former Digitonin at 0.02% may also affect the permeability of the ER membrane or the enzyme activities in the ER. Importantly, higher GPAT/AGPAT activity in 1Δ2Δ3tyΔ cells was only seen after prolonged culture of cells in Doxy, whereas cells grown in sorbitol without Doxy had normal GPAT/AGPAT activity (S5B Fig (1Δ2Δ3tyΔ mutants have normal GPAT and AGPAT activity when not incubated with Doxy) vs. Fig 6C). This finding suggested that it might be the complete depletion of Flc function that induces these acyltransferases or induces a better accessibility of substrates to the enzymes.

**Fig 7:**
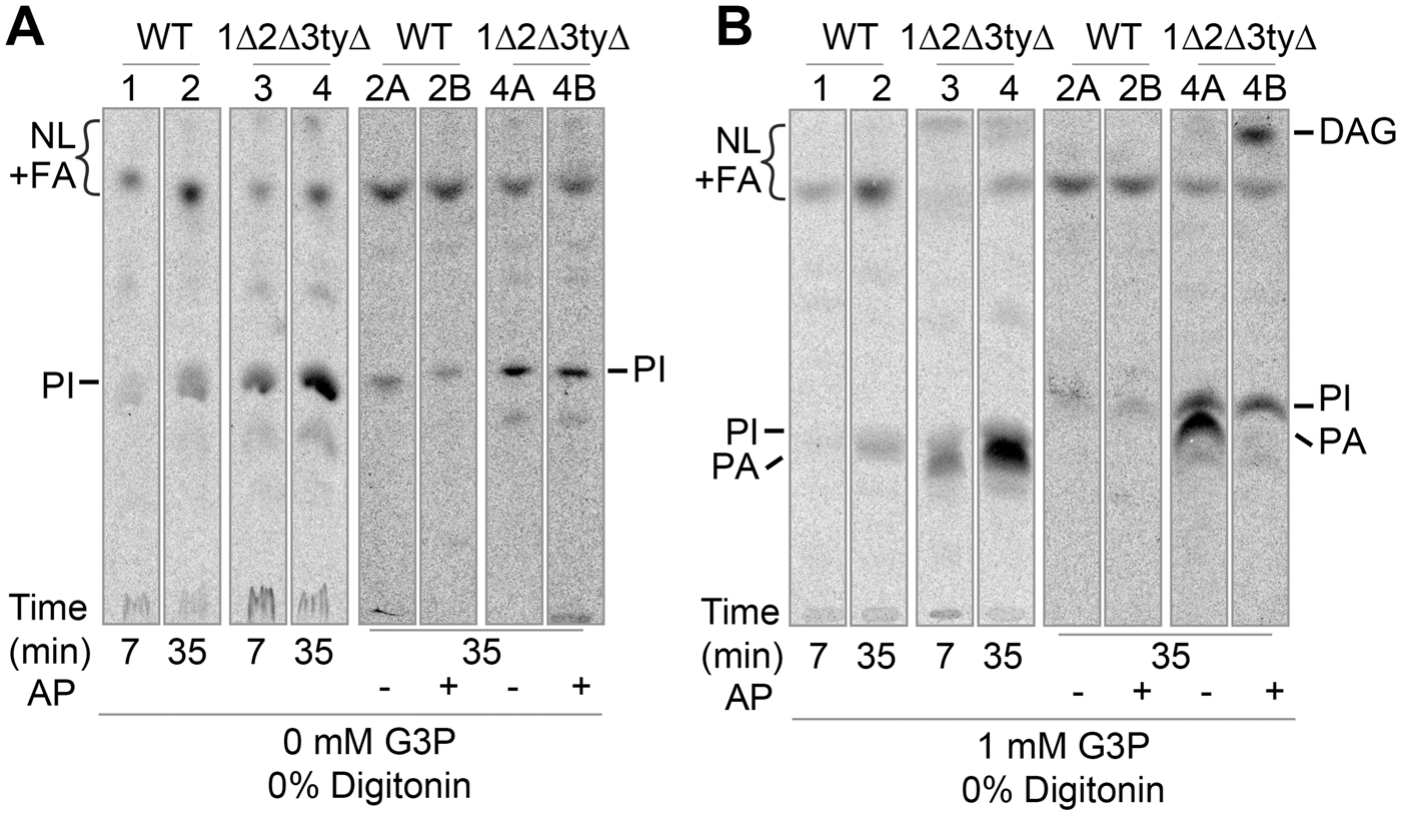
Doxycycline treated 1Δ2Δ3tyΔ mutants have increased GPAT and AGPAT activities. (A, B) WT or 1Δ2Δ3tyΔ cells grown during 16 h with Doxy in 1.4 M sorbitol were incubated with [^3^H]-C16:0-CoA (1 μCi, 10 μM) for the indicated times at room temperature without (A) or with 1 mM G3P (B). PI was distinguished from PA by scraping the silica from lanes 2 and 4 of TLC plates (except the origin), extracting the lipids from silica, splitting samples in two equal halves (2A, 2B and 4A, 4B) and incubating them without (2A, 4A) or with (2B, 4B) alkaline phosphatase (AP). Products were run on TLC and are shown in each panel to the right of the original TLC.

We repeated the experiment of Fig 6A, but labeling permeabilized cells with [^3^H]-C16:0-CoA rather than [^14^C]-G3P. In this setting, in the mere presence of [^3^H]-C16:0-CoA (at 5 mol%), mainly PI was made, but 1Δ2Δ3tyΔ made significantly more PI than WT (Fig 7A). This reaction most certainly means that [^3^H]-C16:0-CoA can enter cells and be used for the acylation of lyso-PI. As soon as [^3^H]-C16:0-CoA was added together with a 100 fold excess of G3P, cells mainly generated PA, again with 1Δ2Δ3tyΔ making much more PA than WT (Fig 7B, lanes 1–4). (As PI and PA had similar migration on TLC, selective transformation of PA into DAG by alkaline phosphatase was used to distinguish PA from PI (Fig 7B, lanes 2B, 4B)). These data indeed argue that the plasma membrane is not an absolute barrier for [^3^H]-C16:0-CoA, nor for G3P if very high concentrations are used, but that in 1Δ2Δ3tyΔ cells the barrier is significantly weaker, for both [^3^H]-C16:0-CoA and G3P as had already been suggested by Fig 6A.

In summary, *flc* mutants exhibit a marked deficit in sphingolipid biosynthesis. The higher GPAT and AGPAT activities at 0.02% as compared to 0.005% Digitonin observed in Doxy treated 1Δ2Δ3tyΔ cells but not WT (Fig 6C and 6D) seem to indicate that Digitonin at 0.02% has a different effect on ER membranes from Doxy treated1Δ2Δ3tyΔ cells, be it that it creates better access of G3P to the ER-based GPATs and AGPATs or better derepression of these enzymes. In this context it is noteworthy that yeast GPATs as well as AGPATs have been found to have their active sites on the lumenal side of the ER by conventional biochemical methods [16,40], but more data are required to support or discredit this somewhat unorthodox hypothesis. While this heightened detergent susceptibility of ER membranes in Doxy treated 1Δ2Δ3tyΔ cells certainly represents no strong argument for a role of Flc proteins in flopping GPLs or acyl-CoA across the ER membrane, it cannot exclude these possibilities either and data ask for more direct experiments.

### Analysis of GPI biosynthesis in *flc* mutants

GPI lipids are built by stepwise addition of sugars to PI: First, N-Acetyl-Glucosamine (GlcNAc) is added to PI, the resulting PI-GlcNAc is then N-deacetylated to PI-GlcN, a FA is attached to the inositol ring to form GlcN-(acyl)PI, which latter then is mannosylated and further modified. While the formation of PI-GlcN is known to occur on the cytosolic side of the ER membrane, the mannosylation occurs in the ER lumen. As *arv1*Δ mutants accumulate GlcN-(acyl)PI, it has been proposed that Arv1, having 4 to 6 predicted TMDs, would serve as a GlcN-(acyl)PI flippase [41]. However, a more recent report demonstrated that Gwt1, the acyltransferase transforming PI-GlcN into GlcN-(acyl)PI has its putative active site on the lumenal side of the ER [22]. This would imply that Arv1 acts after the GPI substrate (PI-GlcN) has already been flipped and suggests that the generation of GlcN-(acyl)PI requires a still unknown flippase other than Arv1, as well as the flipping of acyl-CoA.

**Fig 8:**
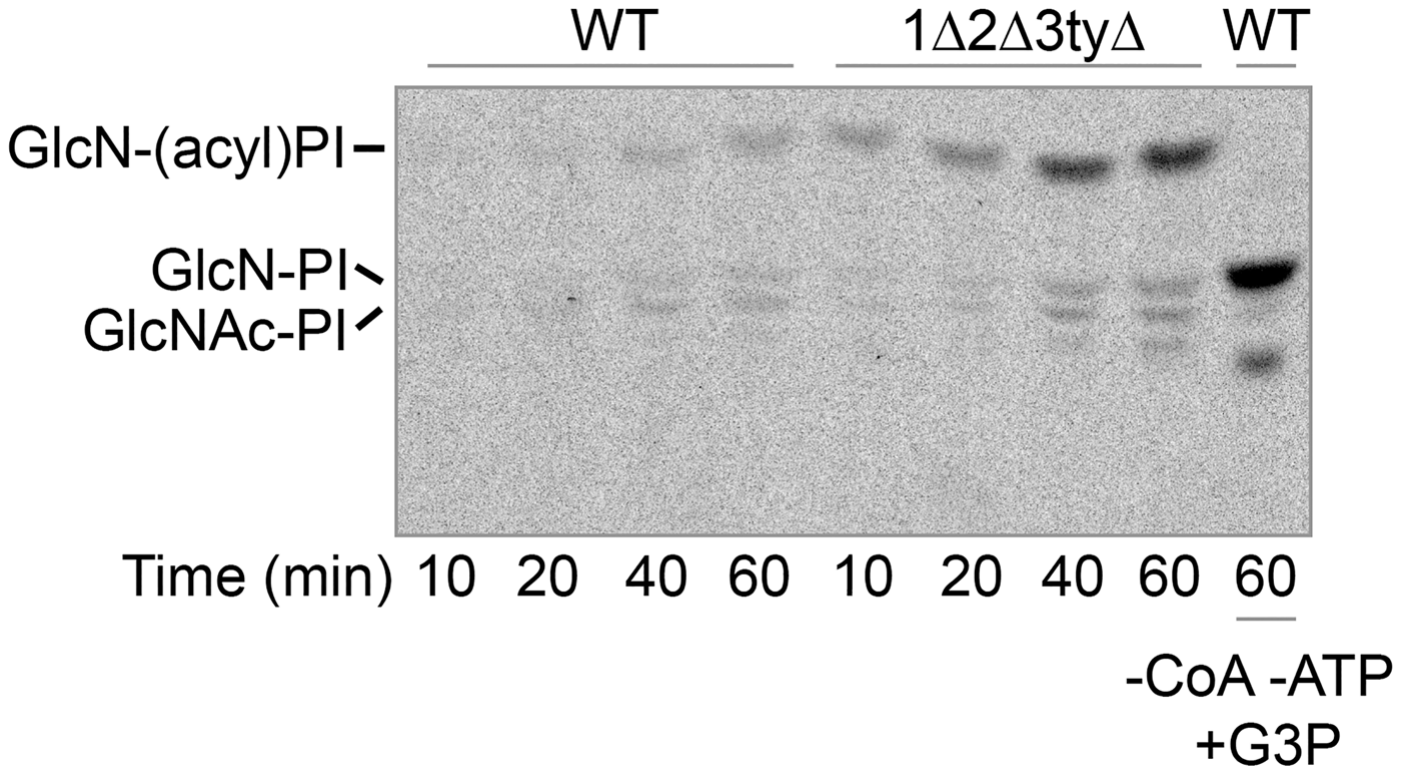
Microsomal GlcN-(acyl)PI synthesis activity in *flc* mutants is increased. Microsomes were produced from WT or 1Δ2Δ3tyΔ cells grown for 12 h in the presence of Doxy and sorbitol, and were labeled using 80 nM UDP-[^3^H]-GlcNAc (4 μCi), 1 mM ATP and 1 mM CoA. In the last lane, to produce GPI intermediates without the acyl chain bound to the inositol moiety, CoA and ATP were omitted and 1 mM G3P was added in order to deplete microsomes of preexisting acyl-CoA. Lipids were extracted and separated by TLC.

As *flc* mutants show a severe cell wall phenotype, characteristic also for mutants with compromised GPI protein biosynthesis, we desired to test whether *flc* mutants have any difficulty in making GlcN-(acyl)PI. We tried to test this by using a microsomal *in vitro* assay of GPI biosynthesis with UDP-[^3^H]-GlcNAc added as the substrate [42]. As shown in Fig 8, microsomes incubated with UDP-[^3^H]-GlcNAc make GlcN-(acyl)PI when they are allowed to make acyl-CoA in presence of added ATP and CoA. Similar results were obtained with permeabilized cells. In all cases Doxy treated 1Δ2Δ3tyΔ cells made significantly more GlcN-(acyl)PI than WT, suggesting that the complete depletion of Flc function induces enzymes for GPI biosynthesis. This finding is reminiscent of the increased GPAT and AGPAT activities in these cells (Figs 5 and 6C and 6D). Once more, results are ambiguous and suggest that the flipping of GlcN-PI and acyl-CoA are normal, but they can’t exclude the possibility that an abnormal permeability or orientation of the ER derived microsomes obviates the need for the corresponding PI-GlcN and acyl-CoA flippases.

### Cst26 introduces C26 fatty acids into lyso-PI

**Fig 9:**
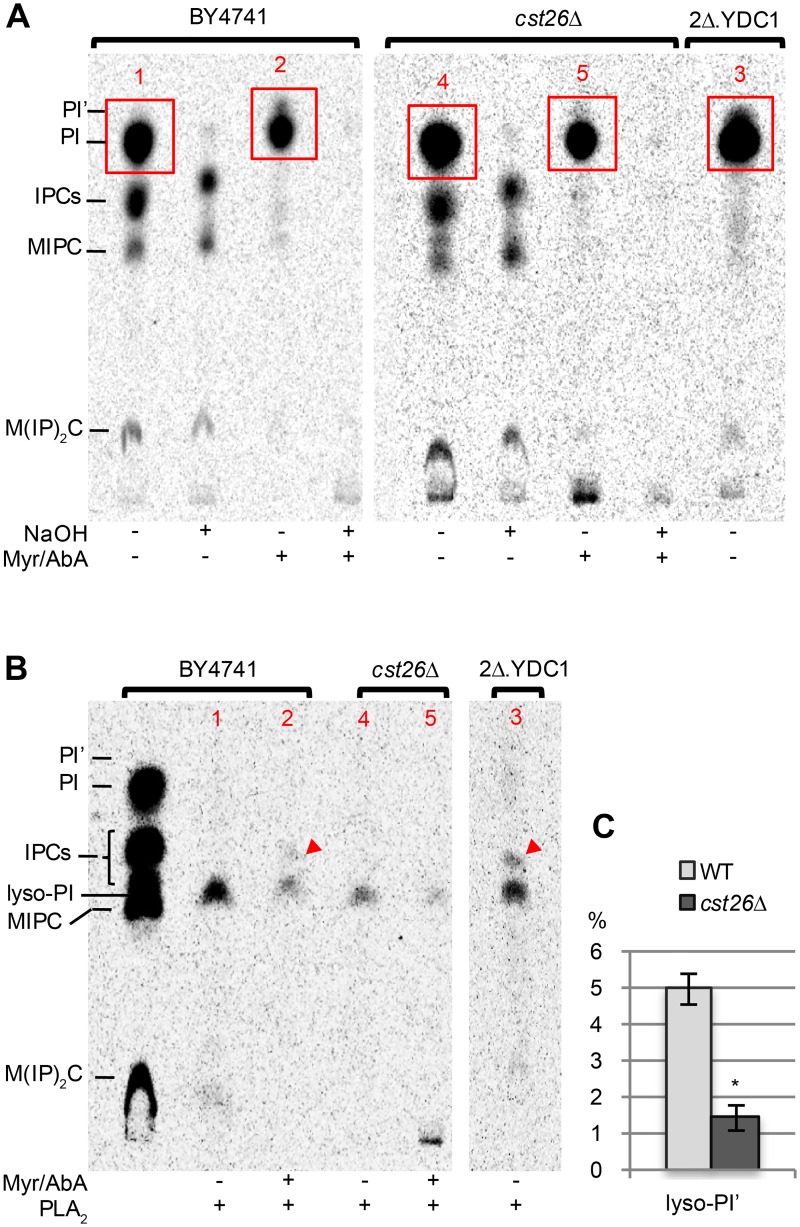
Cst26 introduces VLCFAs into lyso-PI. Cells were grown to exponential phase in SC (2 mg/L of inositol). (A) 3 OD_600_ units of cells were washed and preincubated in inositol-free SC containing or not Myr (40 μg/ml) and AbA (1 μg/ml) for 10 min and then labeled with 10 μCi [2-^3^H]-*myo*-inositol for 4 h at 30°C. Lipids were extracted, deacylated or not with NaOH, desalted and run on TLC in solvent 1. (B) zones in red rectangles in panel A were scraped off the plate, eluted with organic solvent, dried in a rotary evaporator and treated with PLA_2_. Red arrowheads point lyso-PI’. (C) radioscanning was used to quantify radioactivity in lanes containing lipids from Myr/AbA treated cells. Radioactivity was measured in the zone where lyso-PI’ migrates and also in the rest of the lane (except for the origin); lyso-PI’ was then calculated as a percentage of total radioactivity in the lane. In this way, results of the experiment of panel B and a second identical experiment were averaged. * P = 0.002.

Unrelated to the search of lipid flippases, our attention was caught by the very strong negative interactions of *cst26*Δ with *elo2*Δ and *elo3*Δ deletions in MSP- and MSP/C-E-MAPs (S scores between– 9.5 and -11.0). *CST26/PSI1* encodes an acyltransferase that transfers stearic acid (C18:0) from C18:0-CoA onto the *sn*-1 position of lyso-PI [43]. Apart from a further very strong negative interaction with the chitin synthase *CHS1*, *cst26*Δ interacts only with *elo2*Δ and *elo3*Δ, but not *elo1*Δ (see below). *ELO1* allows elongating FAs up to C18:0. *ELO2* and *ELO3* are partially redundant, cannot be deleted simultaneously and are required to further elongate FAs up to C26:0. Each of them is required to make C26:0 in sufficient quantity for the ceramide synthases Lag1 and Lac1, for which C26:0-CoA is the preferred substrate [44]. *Elo2*Δ and *elo3*Δ therefore make markedly reduced amounts of ceramide and mature sphingolipids. However, they grow normally whereas *cst26*Δ *elo3*Δ cells grow less rapidly (S6 Fig (Comparison of growth rates of *elo3*Δ, *cst26*Δ and *elo3*Δ *cst26*Δ cells)). While sphingolipids are essential, their presence is dispensable if, due to a gain of function suppressor mutation in *SLC1*, cells can make PI carrying a very long chain FA in the *sn*-2 position of the glycerol moiety, even if this form of PI accounts for only a tiny fraction of membrane lipids [45–48]. Moreover, in WT cells there exists a natural variety of PI having C26:0 in the *sn*-1 position of glycerol [49], here called PI’, which apparently cannot compensate for the complete loss of sphingolipids. PI’ accounted for <1% of the total PI in WT cells, whereby no C26:0 was detected in other GPLs [43,49–51]. The genetic interaction of *ELO2* and *ELO3* with *CST26* suggested that PI’ helps cells to overcome a relative lack of sphingolipids and that Cst26 may be the still not identified acyltransferase making PI’. As shown in Fig 9A, blocking sphingolipid biosynthesis pharmacologically with myriocin (Myr) and Aureobasidin A (AbA) in BY4741 WT cells leads to the intensification of an [^3^H]-inositol labeled, mild base sensitive band that is less polar than PI, i.e. has a higher mobility on TLC than PI and that we tentatively considered as PI'. The increased synthesis of PI' can be attributed to the accumulation of C26:0-CoA, which in presence of Myr and AbA cannot flow towards its normal destination, ceramides. Biosynthesis of PI’ is significantly weaker in *cst26*Δ (Fig 9A). Ordinary PI in WT cells most often contains a C16:0 in *sn*-1 and a C18:1 in *sn*-2 [43], so that phospholipase A_2_ (PLA_2_) reduces ordinary PI to a lyso-PI with a C16:0 in *sn*-1 (Fig 9B, sample 1). When the [PI + PI’] of WT cells treated with Myr and AbA was subjected to PLA_2_ treatment, an additional lyso-PI with higher TLC mobility was generated (Fig 9B, sample 2). This additional lyso-PI is presumed to represent lyso-PI’, retaining its C26:0 in *sn-*1; this species is also very abundant in *lac1*Δ *lag1*Δ cells (2Δ.YDC1) that lack ceramide synthases [51]. PLA_2_ treatment of [PI + PI’] from *cst26*Δ cells treated with Myr/AbA, generated much less lyso-PI' than treatment of [PI + PI’] from Myr/AbA treated WT cells (Fig 9B, sample 5 vs. 2; Fig 9C). We interpret the data as to say that the bulk of PI carrying C26:0 in *sn*-1 in WT cells is generated by Cst26, and that in *elo2*Δ or *elo3*Δ, Cst26 generates PIs having C18:0, C22:0 or C24:0 in *sn*-1 that may take over a function that is normally exerted by mature sphingolipids. Cst26 thus appears to be an acyl-CoA:lyso-PI acyltransferase that can transfer saturated FAs with 18 to 26 C atoms.

### Cwh43 can modify lyso-GPI anchors and generate abnormal GPI lipids

**Fig 10:**
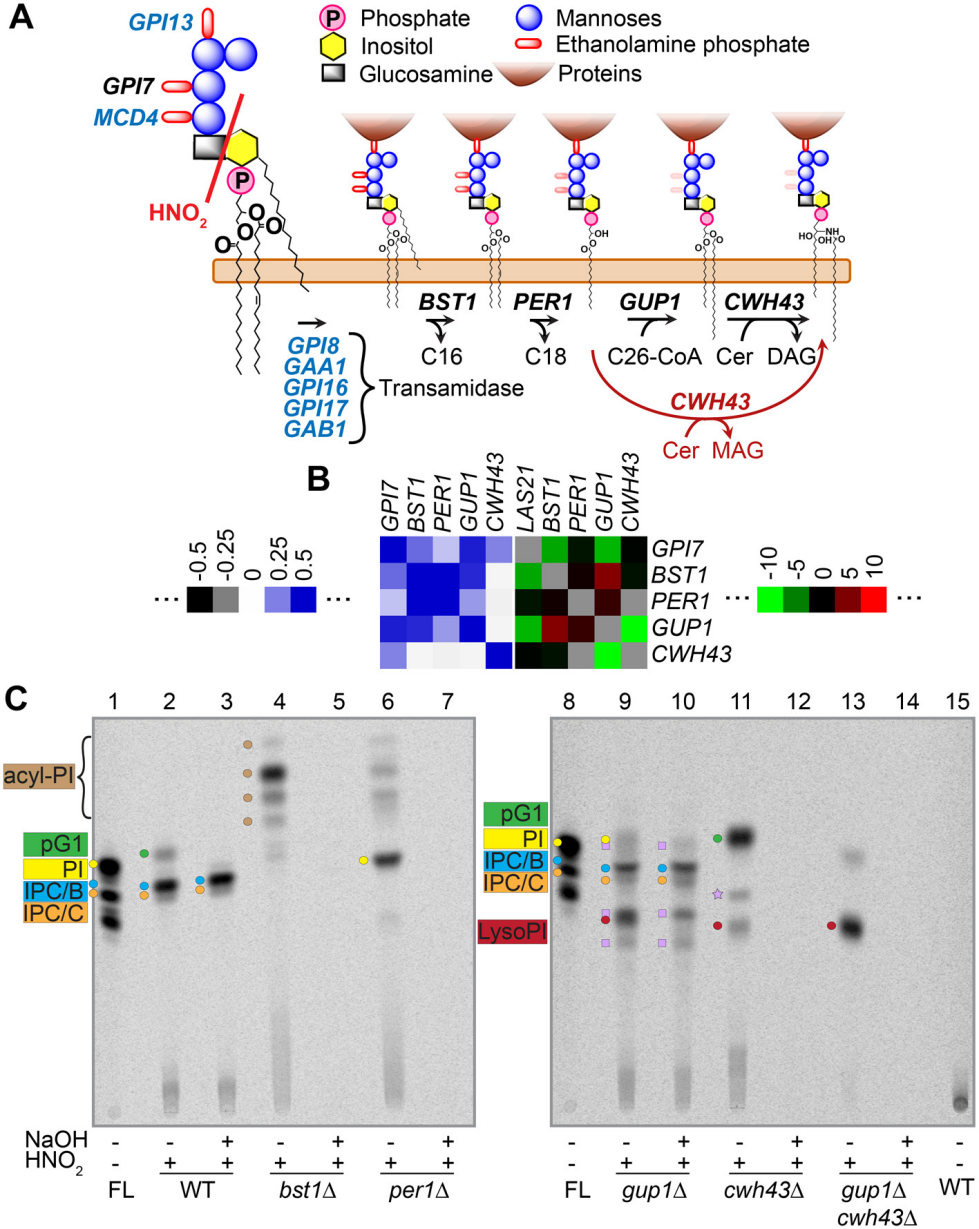
GPI anchor remodeling. (A) the classical model of the lipid remodeling pathway for GPI proteins (black arrows) and a proposed additional route (red arrow)(adapted with permission from Fig 1 of [66]). Essential genes are indicated in blue. MAG = monoacylglycerol. Treatment with HNO_2_ selectively cleaves the glycosidic bond between GlcN and inositol. (B) correlations (left) and S scores (right) of the cluster of GPI anchor remodeling enzymes. Las21 (Gpi7) adds ethanolamine-phosphate to the second mannose (panel A) during GPI lipid biosynthesis and its deletion changes the structure of the GPI anchor. (C) free lipids (FL) and GPI anchor lipid moieties of WT and different remodeling mutants treated or not with nitrous acid (HNO_2_) and mild base (NaOH) as indicated. Lipids were separated on TLC using solvent 3. Light purple squares and stars indicate mild base resistant and mild base sensitive anchor lipids of unknown structure, respectively.

Furthermore, the strongly negative genetic interaction between *gup1*Δ and *cwh43*Δ caught our attention. The lipid moiety of GPI lipids, once attached to proteins, is modified in the ER through so called GPI lipid remodeling reactions. As seen in Fig 10A, the FA on the inositol moiety is removed by Bst1, then the FA in *sn*-2 of the glycerol moiety is removed by the PLA_2_-like Per1, then a C26:0 is transferred from C26:0-CoA onto the *sn*-2 of the lyso-GPI anchor by Gup1, and finally the modified diacylglycerol or phosphatidic acid moiety can be exchanged for a ceramide or a ceramide-phosphate by Cwh43 [52]. All but the last step are prerequisite for an efficient export of GPI proteins out of the ER [15,53–55]. As seen in Fig 10B, the only strong negative genetic interaction in this pathway was between *cwh43*Δ and *gup1*Δ, which did not make sense in the linear pathway depicted in Fig 10A, since the absence of Gup1 in this scheme ought to make the deletion of *CWH43* of no consequence. Indeed, the *gup1*Δ *cwh43*Δ double mutant had a strongly negative S score of– 9.9 and grew more slowly than the single mutants also in liquid culture (S7A Fig (Growth defects of mutants in the right arm of Chromosome II combined with *chs1*Δ)). The same genetic interaction observed in the W303 background recently led to the proposal that the lyso-GPI anchors accumulating in *gup1*Δ may represent suitable substrates for Cwh43 [56] as indicated by the red arrow in Fig 10A. To investigate this in detail, we analyzed the anchor lipids of single and double mutants after metabolic labeling with [^3^H]-*myo*-inositol. As seen in Fig 10C, *bst1*Δ and *per1*Δ cells did not make any base resistant anchor lipids, whereas *gup1*Δ produced lyso-PI and several mild base resistant anchor lipids, two of which comigrated with IPC/B and IPC/C, the typical base resistant anchor lipids of WT, whereas 3 others were not present in WT. Interestingly, not only IPC/B- and IPC/C-type anchors, but also the 3 abnormal mild base-resistant lipids were no more observed in a *gup1*Δ *cwh43*Δ double mutant. The results argue that lyso-GPI anchors indeed are a substrate for Cwh43, as was also proposed by others [56]. In this report it also was proposed that ceramide anchors can be added to GPI-anchors accumulating in *per1*Δ mutants (Fig 10A), but mild base resistance of anchor lipids had not been tested and in our genetic background we can’t see any mild base resistant anchors in *per1*Δ (Fig 10C, lane 7). Moreover, it seems that in the *gup1*Δ background, Cwh43 may transfer also ceramides other than the typical phytosphingosine-C26:0 or phytosphingosine-C26:0-OH giving rise to IPC/B and IPC/C, respectively. Addition of a dihydrosphingosine-C26:0 may account for the most hydrophobic lipid (highest TLC mobility), whereas the utilization of ceramides with shorter or more hydroxylated FAs may explain the appearance of the more polar species. The negative S score of the *gup1*Δ *cwh43*Δ (Fig 10B) argues that the base resistant GPI anchor lipids of *gup1*Δ increase the amount of functional GPI proteins being integrated into the cell wall.

### High profile correlations suggest functions for less well characterized genes

Our E-MAP gene set comprised 99 uncharacterized open reading frames (ORFs). These 99 uncharacterized ORFs however made almost as many significant genetic interactions as the well-characterized genes suggesting that, although still uncharacterized, they are not functionally unimportant or redundant. Some 23 of the 99 non-characterized ORFs were present in 97 gene pairs generating strongly positive correlations (>0.4), whereby in no such pair the partners showed significant genetic interaction with each other (S2D Table). The many high correlations of a deletion in the acyltransferase paralog YDR018c or in the lipase paralog YFL034w with deletions in amino acid permeases suggest that these ORFs may disturb amino acid transport or signaling mediated through such transporters, possibly by disturbing the lipid composition of membranes. Furthermore, in the MSP as well as the MSP/C screen the *ENV10-SSH1* pair was highly correlated (> 0.56) and showed very negative S scores (< - 13). *ENV10* is a not very well characterized gene somehow involved in secretory protein quality control [57], whereas *SSH1* codes for a non-essential homolog of the essential Sec61 translocon subunit of the ER. The very strong *ENV10-SSH1* interaction (not reported in BIOGRID) suggests that Env10, having 4 TMDs and a KXKXX retention signal, may play a role in co-translational protein translocation.

### Deletions in adjacent genes on chromosome II share strong negative interactions with *chs1*Δ and have similar interaction profiles

**Fig 11:**
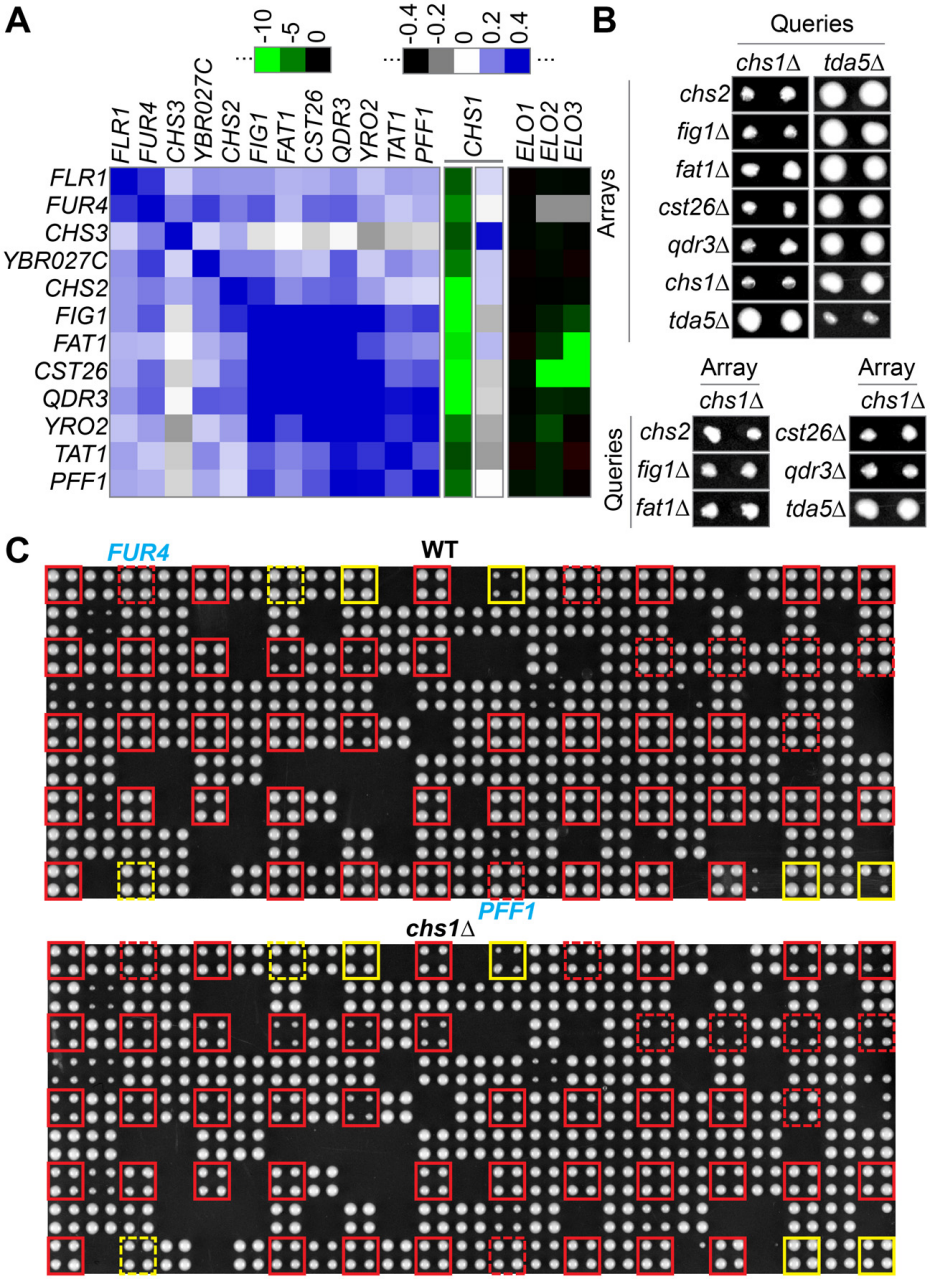
*Chs1*Δ-interacting cluster on chromosome II. (A) correlation and selected interaction heat maps of a series of linked genes on the right arm of Chr. II, deletion of which causes significant negative interactions with *chs1*Δ. Genes are in the same order as they are situated along the chromosome. (B) colonies present on MSP-E-MAP plates after the last selection of double mutants combining deletions in genes indicated on top of column and indicated to the left. As indicated, double mutant colonies are from crosses with *chs1*Δ used as query or being on an array. *Tda5*Δ was added as a neutral control, having an S score of 0.07 with *chs1*Δ. (C) Shows plates from quadruplicate crosses of either a WT query (upper image) or *chs1*Δ query strain (lower image) with an array plate containing many genes from different chromosomes including all the genes from YBR020W to YBR078W on the right arm of Chr. II. All genes in this chromosomal region are boxed, in red if there is a size difference visible to the naked eye between the WT and *chs1*Δ plate, in yellow if there isn't. Boxes are dotted for MSP genes that are part of the E-MAP set and are shown in panel A.

The E-MAP set contained a group of 12 MSP proteins all encoded next to each other in the region between 250’000 and 390’000 bp of the right arm of chromosome II (Chr. II) that presented similar correlations although they are not functionally related (Fig 11A, blue color). These chromosomally clustered positive correlations may be due, at least in part, to uniformly negative genetic interactions of all these genes with *chs1*Δ, all genes having S scores < -3, the genes in the center of the region even <-10 (Fig 11A). Indeed, the colony sizes on the final MSP-E-MAP plates of these pairs on both [query *chs1*Δ x array B of Chr. II] as well as on reciprocal plates were almost the size of the lethal *tda5*Δ *x tda5*Δ control (Fig 11B). The growth rates of the double mutants in liquid and solid media were however normal (S7A and S7B Fig (Growth defects of mutants in the right arm of Chromosome II combined with *chs1*Δ)). To test if negative S-scores appeared also in mutants in that region coding for other proteins than MSPs, we crossed the WT and *chs1*Δ query strains with an array plate containing all the genes of this chromosomal region. This experiment showed that *chs1*Δ combined with a deletion in any gene of this region, whether coding for an MSP or not, had a very negative S score. As shown in Fig 11C, plates showed a regional reduction of colony sizes for genes on Chr. II, whereas the crosses involving genes located on other chromosomes (not boxed), showed no difference of colony sizes between the WT query plate and the *chs1*Δ query plate. In double mutants of *chs1*Δ combined with *chs2-DAmP*, *fig1*Δ, *fat1*Δ, *cst26*Δ, or *qdr3*Δ, we amplified by genomic PCR all genes and intergenic regions starting from *CHS2* up to *QDR3* and found that there were no rearrangements or deletions present apart from the intended single gene deletion. Interestingly, *cst26*Δ is one of the gene deletions sitting in the middle of the chromosomal region where deletions show negative S scores with *chs1*Δ (Fig 11A). As mentioned above, *elo2*Δ and *elo3*Δ also have very negative S scores in combination with *cst26*Δ, similar to *chs1*Δ (Fig 11A). In this case however only very few genes immediately adjacent to *cst26*Δ show a negative S score with *elo2*Δ or *elo3*Δ.

**Fig 12:**
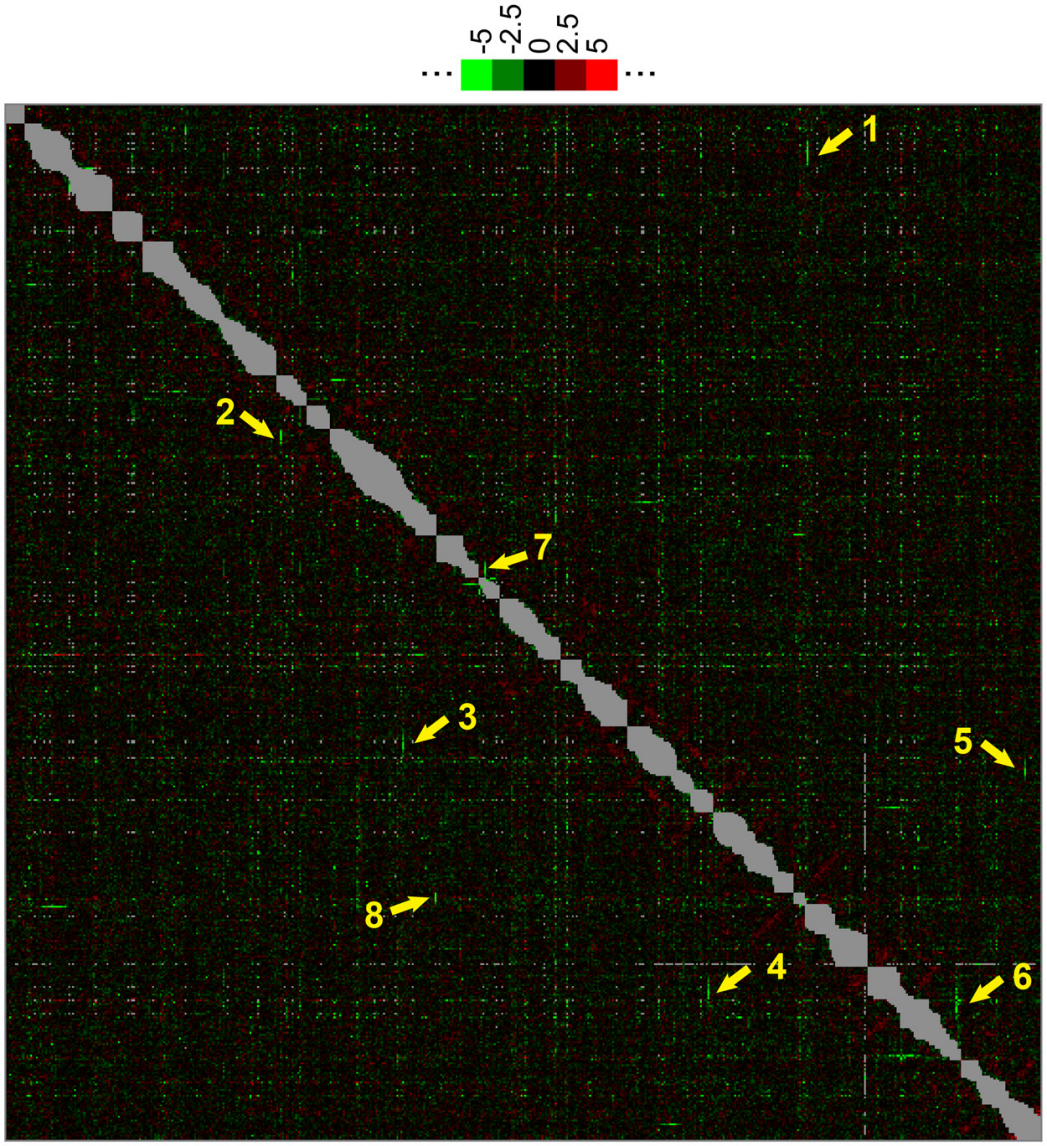
Interaction score heat map with genes ordered according to their chromosomal location. Standard names were replaced by the systematic names in the MSP-E-MAP interaction matrix (S3A Table) and the genes then ordered proceeding from Chr. I to XVI and on each chromosome from the end of the left arm towards the end of the right arm. This rearranged S score matrix (S3C Table) was transformed into the heat map shown here. Arrows point to some short green lines corresponding to a strong negative interaction of a single gene with all MSP set genes in a certain chromosomal region as follows: Arrow 2: *CHO1* interacting with Chr. VII bp 63’048 to 202’273, encompassing *EMC4* (= YGL231C), *OST5*, *VRG4*, *YIP4*, *TPN1*, *YIP5* and *AIM14* (= YGL160W). Arrow 3, *PCP1* interacting with Chr. XII bp 41’280 to 211’933 encompassing *YBT1* (= YLL048C), *GPI13*, *RRT7*, *POM33*, *THI73*, *IZH3* and *SMF3* (= YLR034C). Arrow 4, *TDA5* interacting with Chr. XV bp 114’138 to 242’747 encompassing *WSC3* (= YOL105C), *IZH4*, *YPQ1*, *PHM7*, *YOL079W*, *DSC2*, *RRT8* and *LDS2* (= YOL047C). Arrow 5, *CTR1* (YPR124W) interacting with Chr. XII bp 323’544 to 444’688 encompassing *SUL2* (= YLR092W), *ZRT2*, *NHA1* and *YLR152C*. Arrow 6, *COT1* (YOR316C) at the extreme end of Chr. XV interacting with the centromeric region of the same chromosome (bp 240’204 to 423’732) encompassing *RRT8* (= YOL048C), *LDS2*, *ALG6*, *DFG16*, *AKR2*, *IRC23* and *RSB1* (= YOR049C). Arrow 7, pointing the vertical green line shows *QDR2* interacting with Chr. VIII bp 256’360 to 467’914 encompassing YHR078W, *HXT5*, YHR140W, *CHS7*, *PEX28*, *LAM1* and *SVP26* (= YHR181W). Finally arrow 8, shows *COS6* interacting with Chr. XIV bp 8’330 to 34’696 encompassing *COS1* (= YNL336W), *PFA3*, *LEM3*, *KRE1* and *VNX1* (= YNL321W). This however is a false hit as we found out that *cos6*::*kanMX* in our library is in fact *cos1*::*KanMX*; the confusion arises because the two genes have very similar coding and flanking sequences. 16 well-delimitated grey zones along the diagonal correspond to the negative genetic interactions within each of the 16 chromosomes that were disregarded because of the close linkage of the interacting genes; the size of each zone is proportional to the number of MSPs on that chromosome, not the chromosome.

The phenomenon of regionally concentrated negative interactions shown in Fig 11A is not an isolated phenomenon, since several such regions can easily be identified on a heat map of S scores where the genes are ordered according to their chromosomal location as shown in Fig 12. As the order in this matrix clusters each gene with the genes that sit next to it on the chromosome, all the irrelevant very negative interactions generated by proximity of two deletions on a same chromosome (< 100 kb) and hence marked with grey dots are clustering along the diagonal (Fig 12). Uniform interactions of all deletions in certain chromosomal regions with single deletions on another chromosome or a distant region of the same chromosome appear as short green or red stripes; they are pointed out by numbered arrows, whereby arrow 1 points to the interactions of *chs1*Δ with genes on the right arm of Chr. II discussed above (Fig 11A). Importantly, these chromosomally clustered interactions do not involve the “hyper-interactors” that show interactions throughout the heat map (S8A Fig (Heat maps and main clusters of the MSP-E-MAP)).

We believe that these regionally concentrated negative interactions with a deletion at a distant locus (e.g. *chs1*Δ) are caused by non-declared intergenic suppressor mutations that rescue the growth defect caused by the distant deletions. For example, a gain of function suppressor mutation in *CHS2* present in the *chs1*::*ura3MX* query strain may be present in all crosses of that query except the ones with genes in the vicinity of *CHS2*, where the kanMX-marked array gene will be selected for and the suppressor in *CHS2* is likely to be lost. Such a suppressor mutation in *CHS2* would not exist in *elo2*Δ and *elo3*Δ queries and, if it existed, would not have any genetic interaction with *elo2*Δ and *elo3*Δ strains, explaining the absence of a regional effect around *CST26* in the *elo2*Δ *cst26*Δ and *elo3*Δ *cst26*Δ mutants (Fig 11A). (The strong negative S score of *fat1*Δ *elo3*Δ may not be a neighboring effect but an independent genetic interaction, since Fat1 is the only one of 6 yeast acyl-CoA synthases that can activate very long chain FAs and hence may prepare the substrate for Cst26). Negative genetic interactions appearing in SGA have been utilized before to localize and identify a suppressor mutation in the *SSD1* locus, which suppresses growth effects of mutations in the Cbk1 kinase signaling pathway [58]. In our E-MAP the query strains were generated by swapping the kanMX marker for the ura3MX marker so that suppressors of the array strains had a good chance to be transferred to the query strains (see S3 Text, Materials and Methods). This can explain why the phenomenon for all 8 arrows of Fig 12 was seen symmetrically in both query x array as well as array x query plates. We indeed found that the distant deletions that generated the concerted negative S scores in certain chromosomal regions all had either reduced viability, reduced competitive fitness, a sporulation defect, or reduced respiratory capacity and therefore were susceptible to be overgrown by suppressors.

It is conceivable that such undeclared mutations may cause some noise also in other E-MAP studies using the strategies we used. For instance, in another E-MAP study [59], 6 of the 18 negative interactions of *tda5*Δ were comprised between YOL108cΔ and YOL27cΔ on the left arm of Chr. XV, the same region as pointed by arrow 4 in Fig 12, although none of these negatively interacting deletions were present in our MSP deletion set. Moreover, there are high correlations among functionally unrelated but regionally concentrated genes also in previously published E-MAPs from other groups [60–62].

### Conclusion

We tried to do a chemogenetic screen in order to identify lipid flippases, the existence of which has been postulated since a long time based on microsomal assays and structural studies showing that certain acyltransferases have their active site in the lumen of the ER. No obvious candidates emerged from this, but, in view of the unusual detergent sensitivity and permeability of the plasma and ER membranes of *flc* mutants, a flippase activity of Flc proteins remains a definite possibility, which needs to be pursued by trying to reconstitute Flc proteins into large unilamellar vesicles, e.g. by using and adapting the approaches recently established in our lab [63].

LplT is a lyso-PE transporter of the inner membrane of *E*.*coli* [64]. Deltablasting (http://blast.ncbi.nlm.nih.gov/Blast.cgi) shows some highly significant homologies to 13 yeast genes having > 8% identities covering > 90% of lplT sequence (S2F Table). Ten of them were present in our final array set but none of the 10 was involved in any interaction that got severely aggravated (more negative S score) on Cerulenin. While such homologs remain candidates for GPL flippases, several are localized at the plasma membrane and have well defined transporter functions and the genetic interactions of the others make it unlikely that they would be ER lipid flippases (S2F Table). Another interesting flippase candidate would be the TMEM16 channel homologue *IST2*, which was not present in our deletion library [65].

Our unexpected observation of genetic interactions of certain genes with deletions in an entire chromosomal region may necessitate some additional filtering of the genetic interactions generated in certain E-MAP studies.

## Materials and Methods

All methods have been utilized before and are described in S3 Text and S13 Fig (Titration of Cerulenin to determine its optimal concentration for the MSP/C-E-MAP).

## Supporting Information

S1 Text The MSP- and MSP/C-EMAPs showed the well-known characteristics described for other yeast E-MAPs. (DOCX)

S2 Text Difference between E-MAPs generated in presence and absence of Cerulenin. (DOCX)

S3 Text Reagents strains, plasmids, primers, methods, supplemental figure legends, references used in S3 Text and supplemental figures. (DOCX)

S1 Table (XLSX)

S2 Table (XLSX)

S3 Table (XLSX)

S4 Table (XLSX)

S5 Table (XLSX)

S6 Table (XLSX)

S7 Table (XLSX)

S1 Fig Processing of raw E-MAP data. (TIF)

S2 Fig Reproducibility of correlations of the MSP-E-MAP and MSP/C-E-MAP. (TIF)

S3 Fig Division times of single or combined *flc* mutants. (TIF)

S4 Fig Permeabilization of cells with Digitonin and detection of cytosolic thiol groups with DTNB. (TIF)

S5 Fig 1Δ2Δ3tyΔ mutants have normal GPAT and AGPAT activity when not incubated with Doxy. (TIF)

S6 Fig Comparison of growth rates of *elo3*Δ, *cst26*Δ and *elo3*Δ *cst26*Δ cells. (TIF)

S7 Fig Growth defects of mutants in the right arm of Chromosome II combined with *chs1*Δ. (TIF)

S8 Fig Heat maps and main clusters of the MSP-E-MAP. (TIF)

S9 Fig Enlargement of regions in heat maps of S8A and S8B Fig showing frequent interactions or correlations between genes belonging to two different clusters. (TIF)

S10 Fig Frequency of significant interactions and correlations within and amongst different functional classes of genes. (TIF)

S11 Fig Interdependence of the number of interactions and correlations generated by the MSP-E-MAP. (TIF)

S12 Fig Comparison of E-MAPs with or without Cerulenin. (TIF)

S13 Fig Titration of Cerulenin to determine its optimal concentration for the MSP/C-E-MAP. (TIF)
