# Supplementary material for: Chemogenetic E-MAP in Saccharomyces cerevisiae for Identification of Membrane Transporters Operating Lipid Flip Flop
Source: PLoS Genet. 2016 Jul 27;12(7):e1006160. doi: 10.1371/journal.pgen.1006160 (PMC4962981; doi:10.1371/journal.pgen.1006160)
Supplement: S1 Text — (DOCX) [file pgen.1006160.s001.docx]

**S1_supplemental material**

**for**

**Chemogenetic E-MAP in *Saccharomyces cerevisiae* for identification of membrane transporters operating lipid flip flop**

Hector M. Vazquez*, Christine Vionnet*, Carole Roubaty*, Shamroop Mallela*, Roger Schneiter* and Andreas Conzelmann*^§^

**The MSP- and MSP/C-EMAPs showed the well-known characteristics described for other yeast E-MAPs**

**1) High profile correlations connect functionally related genes.**

In 22 amongst the 68 gene pairs with correlation scores >0.5 in the MSP- or MSP/C-E-MAP (listed in S2C Table), both genes of the pair are involved in N-glycosylation. In a further 6 out of 11 gene pairs involving mitochondrial genes in S2C Table both partners are required for mitochondrial respiration and their S scores are all positive. Other examples of correlations >0.5 concern the functionally related pairs *CHS3-CHS7* required for chitin synthesis and *BST1-PER1* involved in GPI anchor remodeling (S2C Table, see below).

**2) Hierarchical clustering based on less strong profile correlations also clusters functionally related genes together.**

Hierarchical clustering of the 543 genes of the MSP-E-MAP based on the similarity of their correlations (see S3_supplemental material, materials and methods) generated the heat maps of S8 Fig, for which the starting, non-clustered matrices are shown in S4A, S4B Tables, whereas the resulting clustered matrices are shown in S3A and S3B Tables, respectively. The result of hierarchical clustering shown in S8 Fig demonstrates that hierarchical clustering brings together genes working for similar functions even if their profile correlations are lower than 0.4.

*Cluster 1 of S8A Fig groups together genes for protein glycosylation and cell wall biosynthesis.* Cluster 1 in S8A Fig contains genes involved in N-glycosylation, which interact negatively with each other, causing the green coloring next to the diagonal. Blow up of this cluster (S8C Fig) shows that genes are subdivided in two sub-clusters consisting of a) those that build the dolichol-linked oligosaccharide (*ALG8* to *DIE2*) and b) the subunits of the oligosaccharyltransferase (OST)(*OST3* to *SWP1*) that transfers the oligosaccharide to nascent substrate proteins in the ER as described before [1]. As expected, deletions in the former sub-cluster are epistatic and result in no or positive genetic interactions between each other, but deletions in the former sub-cluster interact very negatively with deletions of OST subunits of the second sub-cluster. This recapitulates the well-known fact that the defect caused by a crippled OST is enhanced, when it has to transfer a crippled oligosaccharide. Within the OST deletions, some pairs are neutral, but some result in very negative interactions as well. Cluster 1 also contains *GUP1* and *KRE1,* required respectively for GPI protein remodeling and β-glucan biosynthesis, processes which, together with N-glycosylation, collaborate for cell wall integrity (CWI) [2,3]. *GUP1* and *KRE1* however are the only genes of cluster 1 to also have a high number of negative and positive interactions outside of cluster 1 and throughout the heat-map*,* suggesting that they have a greater impact on cell growth than N-glycosylation. Several of the genes involved in GPI or cell wall glucan biosynthesis of cluster 1 (*GUP1, LAS21, ARV1* and *KRE1*) interact also negatively with *CCH1, CHS3, CHS7,* *ERG3,* and *OST6* of cluster 4 (see below)*,* which are equally required for CWI (S8A Fig, box 1γ). Thus, genes required for CWI are split up between clusters 1 and 4.

*Cluster 5 of S8A Fig contains mitochondrial genes, which frequently interact with genes in other functional categories.* The diagonal of S8A Fig also highlights cluster 5, most genes of which are required for mitochondrial respiration. These genes interact positively with each other, as expected, but they also interact with a great number of other genes far from the diagonal, highlighted by boxes 1δ, 5η, and 5θ. Thus, genes of cluster 5 contain frequent interactions with several genes of cluster 1 required for cell wall integrity (CWI) (*BST1, GUP1, KRE1, ARV1, ALG6*)(box 1δ). The positive interactions of box 1δ may suggest that the slow growth phenotype of respiration deficient mutants (*PAM17, MGR2, TIM17*) makes cell wall biosynthesis less important, but this seems to be the case only in certain conditions because other non-respiring mutants of cluster 5 don't show these positive interactions, and since *GPI2* and *GPI13*, two other genes affecting cell wall biosynthesis, interact negatively with cluster 5 genes*.* Intriguingly, most genes in the mitochondrial cluster 5 show strong negative interactions with the hyper-interactor *SUR1* (=*CSG1*), required for mannosyl-phosphorylinositolceramide biosynthesis and with *AUS1*, a lipid flippase at the plasma membrane*,* although none of these interactions is reported in BIOGRID (S9 Fig).

**3) Hierarchical clustering often brings together functionally related genes, which amongst themselves show no major genetic interactions but only correlations.**

Such is the case for clusters 2 – 4 and 6, which appear as blue regions along the diagonal in S8B Fig, but are not very colorful in S8A Fig and S8C Fig. They get clustered together because of the similarity of their genetic interactions with other genes, reflected by strong blue color far from the diagonal.
*Cluster 2* contains the *DFG16*/*RIM9* pH sensor complex, whereby deletion of either one destroys the sensing function. This results in the expected picture of high correlation and also a positive genetic interaction of *dfg16∆* and *rim9∆* mutations (enlarged in S8C Fig).
*Cluster 3* contains many transporters but also genes with other functions and interacts quite negatively with the mitochondrial respiration genes of cluster 5 (S8A Fig, box 5η, S9 Fig).
*Cluster 6* of S8B Fig seems to be functionally heterogeneous, but appears as the epicenter of a larger cluster grouping genes, positively correlated not only amongst themselves but also with groups far from the diagonal (S8B Fig, boxes α, 2ζ, 5θ). Only boxes β and 5θ, i.e. the *ELO2/CCC2/FTR1* microcluster and the mitochondrial respiration genes of cluster 5 interact genetically with the cluster 6 genes (S8A Fig). Also visible in S8B Fig, pairs combining genes from cluster 6 with genes from clusters 3 or 4 show negative correlations (grey zones).

**4) Interactions amongst different functional classes**.

Instead of letting the hierarchical clustering bring together functionally related genes, we inverted the process by first grouping our 543 E-MAP genes into 11 classes according to the biological process annotations as described in S1 Table and reflected in the color codes of S8C Fig and then interrogating the data for the frequency of interactions and correlations within classes and between different classes. S10A Fig shows a high tendency for negative interactions and positive correlations within the group of genes acting in protein maturation/targeting (purple). To a lesser degree this also is true for genes involved in biosynthesis of the cell wall (red). Protein maturation and cell wall biosynthesis genes also strongly interact with each other in that there are frequent negative interactions combined with positive correlations between genes of these two classes S10A Fig. Within the class of mitochondrial genes (cyan) there is a tendency to have positive genetic interactions and positive correlations reflected in S8A Fig by cluster 5. Corresponding boxes in S8 Fig mirror all these elevated interaction and correlation frequencies.

**5) Statistical analysis of E-MAPs.**

Not having performed E-MAPs before, we have undertaken some statistical analysis of our E-MAPs, which were certainly done but not necessarily reported for previous E-MAPs. One approach was to compare the 543 genes of the E-MAP set with each other rather than compare pairs of genes to each other. As can be seen from S11A and S11D Fig, in the MSP-E-MAP, genes generated an average of 1.6 positive and 1.1 negative significant interactions and of 1.85 positive and 1.1 negative significant correlations. Yet, some genes were found to be involved in significant interactions much more frequently than others, with 5 of the 8 top runners being involved in lipid biosynthesis (*GUP1, SAC1, ELO3, SUR1 (=CSG1), ERG3*), confirming findings of previous high throughput reports [1,4-6] (S11A Fig, S5 Table). These genes are visible on S8A Fig as colored lines, especially if their interactions are not distributed randomly but segregate into regions where positive, others where negative interactions predominate, as is the case for *GUP1*/*KRE1*. Amazingly, *ILM1,* one of the most interactive genes (S11A Fig), has not been functionally characterized. The *ELO3/SAC1* microcluster is not part of cluster 1 but forms multiple positive and negative interactions with *ALG* genes of cluster 1 as can be seen in the enlarged regions of S8C Fig (and throughout the heat map), pointing not yet understood functional interactions between lipid biosynthesis and N-glycosylation. Similarly, the microcluster of *ELO2/CCC2/FTR1* with the later two transporting copper and iron, respectively, is negatively interacting with a total of 19 genes (P<0.005), 15 of which are clustered in boxes 2ε and β. The existence of such “hyper-interactors” is also visible when one considers the sum of the significant S scores rather than the number of significant interactions each gene deletion is generating (S11B Fig). Moreover, there is a positive correlation between the number of interactions and the number of correlations each gene generates (S11F Fig), the strongest correlation being the one between the number of negative interactions and the number of positive correlations (S11C Fig). In spite of these tendencies there were 38 genes making no genetic interactions of which 25 still made correlations, and there also were 125 genes not making any correlations but only interactions (S11E Fig).

**References for S1_supplemental materials**

1. Schuldiner M, Collins SR, Thompson NJ, Denic V, Bhamidipati A, Punna T, et al. Exploration of the Function and Organization of the Yeast Early Secretory Pathway through an Epistatic Miniarray Profile. Cell. 2005;123: 507–519. doi:10.1016/j.cell.2005.08.031

2. Bosson R, Jaquenoud M, Conzelmann A. GUP1 of Saccharomyces cerevisiae encodes an O-acyltransferase involved in remodeling of the GPI anchor. Mol Biol Cell. 2006;17: 2636–2645. doi:10.1091/mbc.E06-02-0104

3. Boone C, Sommer SS, Hensel A, Bussey H. Yeast KRE genes provide evidence for a pathway of cell wall beta-glucan assembly. J Cell Biol. 1990;110: 1833–1843.

4. Jonikas MC, Collins SR, Denic V, Oh E, Quan EM, Schmid V, et al. Comprehensive characterization of genes required for protein folding in the endoplasmic reticulum. Science. 2009;323: 1693–1697. doi:10.1126/science.1167983

5. Costanzo M, Baryshnikova A, Bellay J, Kim Y, Spear ED, Sevier CS, et al. The genetic landscape of a cell. Science. American Association for the Advancement of Science; 2010;327: 425–431. doi:10.1126/science.1180823

6. Hoppins S, Collins SR, Cassidy-Stone A, Hummel E, Devay RM, Lackner LL, et al. A mitochondrial-focused genetic interaction map reveals a scaffold-like complex required for inner membrane organization in mitochondria. The Journal of Cell Biology. 2011;195: 323–340. doi:10.1083/jcb.201107053
