# Supplementary material for: Chemogenetic E-MAP in Saccharomyces cerevisiae for Identification of Membrane Transporters Operating Lipid Flip Flop
Source: PLoS Genet. 2016 Jul 27;12(7):e1006160. doi: 10.1371/journal.pgen.1006160 (PMC4962981; doi:10.1371/journal.pgen.1006160)
Supplement: S2 Text — (DOCX) [file pgen.1006160.s002.docx]

**S2_supplemental material**

**for**

**Chemogenetic E-MAP in *Saccharomyces cerevisiae* for identification of membrane transporters operating lipid flip flop**

Hector M. Vazquez*, Christine Vionnet*, Carole Roubaty*, Shamroop Mallela*, Roger Schneiter* and Andreas Conzelmann*^§^

**Difference between E-MAPs generated in presence and absence of Cerulenin**

As shown in S12A Fig, the majority of genetic interactions were quite similar whether or not Cerulenin was present during the last selection, and the correlation between S scores in MSP- and MSP/C-E-MAP was even higher than between replicates 1 and 2 (Fig 1F, 1G). Yet, as seen in S12A Fig, a minority of interactions became less sick (red dots), or sicker on Cerulenin (green dots). Similarly, when comparing the performance of individual genes in the E-MAP without and with Cerulenin, the numbers of significant positive and negative interactions and of positive correlations were positively correlated and in particular the number of negative interactions was highly correlated (R = 0.9). Strong interactions were very reproducible in MSP- and MSP/C-E-MAPs (Fig 1F, 1G) suggesting that also the changes leading to appearance or disappearance of such strong S scores on Cerulenin were significant. Gene pairs showing such strong changes are listed in S2E Table and are 4 fold enriched in genes required for lipid biosynthesis and >6 fold in genes for GPI/cell wall biosynthesis. *Elo3∆, sac1∆, per1∆* and *bst1∆* were present in both, pairs having much lower as well as pairs having much higher S scores on Cerulenin (S2E Table).
