# Supplementary material for: Chemogenetic E-MAP in Saccharomyces cerevisiae for Identification of Membrane Transporters Operating Lipid Flip Flop
Source: PLoS Genet. 2016 Jul 27;12(7):e1006160. doi: 10.1371/journal.pgen.1006160 (PMC4962981; doi:10.1371/journal.pgen.1006160)
Supplement: S3 Text — (DOCX) [file pgen.1006160.s003.docx]

**S3_Supplementary material**

**for**

**Chemogenetic E-MAP in *Saccharomyces cerevisiae* for identification of membrane transporters operating lipid flip flop**

Hector M. Vazquez*, Christine Vionnet*, Carole Roubaty*, Shamroop Mallela*, Roger Schneiter* and Andreas Conzelmann*^§^

**Lists of strains, plasmids and primers**

**Yeast strains**

| **Strain name** | **Genotype** | **Source** |
| --- | --- | --- |
| BY4741 | *MAT*a *his3Δ1 leu2Δ0 met15Δ0 ura3Δ0* | Euroscarf |
| BY4742 | *MAT*α *his3Δ1 leu2Δ0 lys2Δ0 ura3Δ0* | Euroscarf |
| Euroscarf deletion library (Array) | *MAT*a *his3Δ1 leu2Δ0 met15Δ0 ura3Δ0 xxxX∆::KanMX4* | Euroscarf |
| DAmP library (Array) | *MAT*a *his3Δ1 leu2Δ0 met15Δ0 ura3Δ0 xxxX-DAmP-KanMX4* | [1] |
| y8205 | *MAT*α *can1∆::STE2pr-Sp_his5 lyp1∆::STE3pr-LEU2 his3∆1 leu2∆0 ura3∆0* | C. Boone |
| ura3::caURA3MX4 | *MAT*α *can1∆::STE2pr-Sp_his5 lyp1∆::STE3pr-LEU2 his3∆1 leu2∆0 ura3::caURA3MX4* | [2] |
| MSP-E-MAP queries | *MAT*α *can1∆::STE2pr-Sp_his5 lyp1∆::STE3pr-LEU2 his3∆1 leu2∆0 ura3∆0 yyyY∆::caURA3MX4* | This study |
| MSP-E-MAP double mutants | *MAT*a *can1∆::STE2pr-Sp_HIS5 lyp1∆::STE3pr-leu2 his3∆1 leu2∆0 ura3∆0 xxxX∆::KanMX4* yyyY∆::caURA3MX4 | This study |
| *flc1∆* | *MAT*α *can1∆::STE2pr-Sp_his5 lyp1∆::STE3pr-LEU2 his3∆1 leu2∆0 ura3∆0 flc1∆::caURA3MX4* | This study |
| *flc2∆* | *MAT*α *can1∆::STE2pr-Sp_his5 lyp1∆::STE3pr-LEU2 his3∆1 leu2∆0 ura3∆0 flc2∆::NatMX4* | This study |
| *flc3∆* | *MAT*a *his3Δ1 leu2Δ0 met15Δ0 ura3Δ0 flc3∆::KanMX4* | Euroscarf |
| *yor365c∆* | *MAT*a *his3Δ1 leu2Δ0 met15Δ0 ura3Δ0 yor365c∆::HisMX4* | This study |
| *flc1∆ flc2∆* | *MAT*α *can1∆::STE2pr-Sp_his5 lyp1∆::STE3pr-LEU2 his3∆1 leu2∆0 ura3∆0 flc1∆::caURA3MX4 flc2∆::NatMX4* | This study |
| *flc1∆ flc3∆* | *MAT*α *can1∆::STE2pr-Sp_his5 lyp1∆::STE3pr-LEU2 his3∆1 leu2∆0 ura3∆0 flc1∆::caURA3MX4* *flc3∆::KanMX4* | This study |
| *flc1∆ yor365c∆* | *MAT*α *can1∆::STE2pr-Sp_his5 lyp1∆::STE3pr-LEU2 his3∆1 leu2∆0 ura3∆0 flc1∆::caURA3MX4 yor365c∆::HisMX4* | This study |
| *flc2∆ flc3∆* | *MAT*α *can1∆::STE2pr-Sp_his5 lyp1∆::STE3pr-LEU2 his3∆1 leu2∆0 ura3∆0 flc2∆::NatMX4 flc3∆::KanMX4* | This study |
| *flc2∆ yor365c∆* | *MAT*α *can1∆::STE2pr-Sp_his5 lyp1∆::STE3pr-LEU2 his3∆1 leu2∆0 ura3∆0 flc2∆::NatMX4 yor365c∆::HisMX4* | This study |
| *flc3∆ yor365c∆* | *MAT*a *his3Δ1 leu2Δ0 met15Δ0 ura3Δ0 flc3∆::KanMX4 yor365c∆::HisMX4* | This study |
| *flc1∆ flc2∆ tetO-FLC3* | *MAT*α *can1∆::STE2pr-Sp_his5 lyp1∆::STE3pr-LEU2 his3∆1 leu2∆0 ura3∆0 flc1∆::caURA3MX4 flc2∆::NatMX4 tetO2-FLC3-KanMX4* | This study |
| *flc1∆ flc2∆ yor365c∆* | *MAT*α *can1∆::STE2pr-Sp_his5 lyp1∆::STE3pr-LEU2 his3∆1 leu2∆0 ura3∆0 flc1∆::caURA3MX4 flc2∆::NatMX4 yor365c∆::HisMX4* | This study |
| *flc1∆ flc3∆ yor365c∆* | *MAT*α *can1∆::STE2pr-Sp_his5 lyp1∆::STE3pr-LEU2 his3∆1 leu2∆0 ura3∆0 flc1∆::caURA3MX4 flc3∆::KanMX4 yor365c∆::HisMX4* | This study |
| *flc2∆ flc3∆ yor365c∆* | *MAT*α *can1∆::STE2pr-Sp_his5 lyp1∆::STE3pr-LEU2 his3∆1 leu2∆0 ura3∆0 flc2∆::NatMX4 flc3∆::KanMX4 yor365c∆::HisMX4* | This study |
| *flc1∆ flc2∆ tetO-FLC3 yor365c∆* 1∆2∆3ty∆ | *MAT*α *can1∆::STE2pr-Sp_his5 lyp1∆::STE3pr-LEU2 his3∆1 leu2∆0 ura3∆0 flc1∆::caURA3MX4 flc2∆::NatMX4 tetO2-FLC3-KanMX4 yor365c∆::HisMX4* | This study |
| *chs2-DAmP* | *MAT*a *his3Δ1 leu2Δ0 met15Δ0 ura3Δ0 chs2-DAmP-KanMX4* | [1] |
| *bst1∆* | *MAT*α *can1∆::STE2pr-Sp_his5 lyp1∆::STE3pr-LEU2 his3∆1 leu2∆0 ura3∆0 bst1∆::caURA3MX4* | This study |
| *per1∆* | *MAT*α *can1∆::STE2pr-Sp_his5 lyp1∆::STE3pr-LEU2 his3∆1 leu2∆0 ura3∆0 per1∆::caURA3MX4* | This study |
| *gup1∆* | *MAT*α *can1∆::STE2pr-Sp_his5 lyp1∆::STE3pr-LEU2 his3∆1 leu2∆0 ura3∆0 gup1∆::caURA3MX4* | This study |
| *cwh43∆* | *MAT*α *can1∆::STE2pr-Sp_his5 lyp1∆::STE3pr-LEU2 his3∆1 leu2∆0 ura3∆0 cwh43∆::caURA3MX4* | This study |
| *gup1∆ cwh43∆* | *MAT*α *can1∆::STE2pr-Sp_his5 lyp1∆::STE3pr*-*LEU2 his3∆1 leu2∆0 ura3∆0 cwh43∆::caURA3MX4 gup1∆::KanMX4* | This study |

**Plasmids**

| Name | Type and markers | Source |
| --- | --- | --- |
| pAG60 | caURA3MX4 | [3] |

**Primers for PCR**

| Number | Purpose | Sequence (5’-3’) |
| --- | --- | --- |
| 1388 | Forward primer to swap KanMX4 to caURA3MX4 | ACATGGAGGCCCAGAATACCC |
| 1389 | Reverse primer to swap KanMX4 to caURA3MX4 | CAGTATAGCGACCAGCATTCAC |

**Materials and methods**

*Strains and reagents.* Strains, plasmids and primers used are listed above. Cells were grown at 30°C on either YPD (1% yeast extract, 2% peptone, 2% glucose) or on synthetic complete (SC) medium (Yeast nitrogen base with (YNB) or without ammonium sulfate (YNB - A.S.)) plus dropout mix (amino acids, inositol, etc.) plus 2% glucose and plus 0.1% L-glutamic acid, when ammonium sulfate was omitted. Media for robotically crossing strains were the ones described in [4]. Sorbitol, glucose, G418, and Canavanine were from ForMedium (UK). Amino acids, Doxycycline, Thialysine, Chitinase from *Trichoderma viride*, Acyl-Coenzyme A synthetase from *Pseudomonas sp.,* Glycerol-3-phosphate, DTNB, Calcofluor white, Caspofungin diacetate, Tunicamycin from a *streptomyces* species, Triton TX-100, Digitonin, inositol, NaN_3_, NaF and buffer salts were from Sigma. Coenzyme A (=CoA) trilithium salt was from Merck. [^3^H]-*myo*-inositol, [^3^H]-palmitic acid and [^14^C]-Glycerol-3-phosphate were from ANAWA Trading SA (Switzerland). [^3^H]-palmitoyl-CoA was synthesized as previously described [5]. Cerulenin was from Enzo Life Science AG (Switzerland), Zymolyase-20T from Seikagaku (Japan).

*Preparation of strains for E-MAP Analysis*. A list of 758 genes coding for proteins with two or more transmembrane regions, as predicted by the TMHMM algorithm, was obtained from the Comprehensive Yeast Genome Database (CYGD) (<http://mips.helmholtz-muenchen.de/genre/proj/yeast/>) (S6 Table). Of this list 579 deletion mutants were present in our EUROSCARF deletion library and 48 in our DAmP strain collection harboring hypomorphic alleles of essential genes [1]. *Ybr219c∆* and *slc1∆* were also included in our E-MAP strain collection. These 629 strains were grown in 200 µg/ml G418 at 24°C and frozen in 96-well plates containing liquid YPD supplemented with 15% glycerol. In the following, “query strains” designate strains harboring the two "magic markers" *can1∆::STE2pr-Sp_his5* and *lyp1∆::STE3pr-LEU2* plus a *xxxX::caURA3MX4* deletion; “array strains” designate *CAN1 LYP1* *yyyY::kanMX* strains [6,7]. To obtain such query strains, we used the *MAT***a** array library from EUROSCARF and replaced the kanMX4 marker by the *caURA3MX4* marker containing the *URA3* of *Candida albicans* [3]. The swapping element was PCR amplified from the plasmid pAG60 with primers n°1388 and n°1389 and used to transform the array collection in microtiter plates with the LiAc/SS carrier DNA/PEG method as previously described [8]. After three consecutive rounds of transformations finally all the mutants had become uracil prototroph and were streaked on agar plates; then single colonies were re-streaked in parallel on SC without uracil and YPD with G418. 590 of 629 mutants had lost G418 resistance and gained uracil prototrophy. The 590 uracil prototroph *MAT*a strains were mated with strain y8205 containing the magic markers, sporulated and *MAT*α or *MAT*a haploid progeny containing *can1∆::STE2pr-Sp_his5* *lyp1∆::STE3pr-LEU2* plus a *xxxX::caURA3MX4* deletion was selected, thus creating the query strain set. 583 strains were obtained once in the *MAT***a** and once in the *MAT***α** background*.* The two query sets were streaked again on agar plates, single colonies were randomly chosen and used to inoculate liquid YPD containing plates in 96-well format to be frozen at -80°C with 15% glycerol until use for E-MAPs.

To control for correct gene deletions, *MAT*a queries were robotically mated with the identical gene mutations in kanMX format, but coming from a different EUROSCARF deletion library than the one used to generate the query strains. After sporulation and selection of haploid double mutants, only few strains grew at the last selection. Growth suggested a false gene deletion; parents of growing strains were therefore controlled by PCR, replaced if necessary and controlled again by PCR. Thus, two sets of query strains, one *MAT*a and one *MAT*α, each containing 539 deletion and 44 hypomorphic mutants (DAmP), were thus prepared.

*E-MAP Analysis*

The genetic screen was done exactly as previously described [4], except that the *MAT*α query strains contained the *caURA3MX4* instead of *NATMX4* marker, the single mutant selection plates were replicated in parallel onto normal double mutant selection plates and on the same but supplemented with 0.4 µg/ml Cerulenin; these double mutant selection plates were incubated at 30°C for 24 h before pictures of the plates were taken. Cerulenin was titrated by robotically replicating the E-MAP *xxx::kanMX* array collection on plates with different concentrations of Cerulenin. As judged by visual inspection of plates, at concentrations of 0.4 µg/ml, most strains were still growing normally (S13 Fig). For the MSP-E-MAPs we used an array of 629 mutants in duplicates arranged horizontally with a maximal density of 1536 colonies per plate. Each one of the 583 queries was crossed with such an array plate. Color pictures were taken using a Nikon D90 (12.3 megapixels), focal length = 105 mm, F-number = 8, ISO = 200, mounted in a KAISER camera stand with two fixed TC-L lamps (36W). The plates were put on top of a black surface to have a uniform black background.

Plate pictures were cropped using Photoshop, treated with the HT Colony Grid Analyzer program and the resulting files processed using the E-MAP toolbox. All these software are described in [4,9] and can be downloaded from the web. The settings in the E-MAP toolbox for the processing of data were *2^nd^ order surface* for position artifacts, *Parzen estimates* for normalization and control size estimations, *0.4* as threshold for removing frequently missing strains, *10* and *1000*, respectively, for minimum and maximum size threshold.

All P-values used for the selection of significance thresholds were calculated separately for negative and positive values throughout and were computed using the (one tailed) probability density calculation of the Excel NORM.DIST function. Unless indicated otherwise, P values were not adjusted, e.g. by Bonferroni or Benjamini-Hochberg corrections. Thus, we considered as significant those genetic interactions having unadjusted P-values of <0.005, correlations having P-values <0.05, because these significance levels appeared to be similar to the thresholds used in other E-MAP studies. Indeed, Bonferroni correction removed many interactions and corrections that made biological sense. E.g. the clear dependence between correlations and mean S scores of Fig 2 would be between non-significant correlations and S scores. Significance levels based on Bonferroni-corrected P values (P<0.05) are however indicated by dotted lines in Fig 2 and interactions and correlations being signficant under these more stringent criteria are marked in S2B Table.

*Data clean up*

As shown in S6 Table, from a total of 629 strains present in the array and 583 strains present in the queries, 17 and 16 strains, respectively, were eliminated because of their proximity to *CAN1* or *LYP1* (< 100 kbp up or downstream) 10 and 21 strains, respectively, were eliminated due to mating, sporulation or germination problems, 42 mutants were eliminated in both queries and arrays because in systematic visual inspection of the plates they were considered to be noisy, showed very inconstant scoring or colony sizes and counted more than 10 interactions with an S score difference ≥⏐5⏐between the unaveraged S scores of the reciprocal [query A x array B] and [query B x array A] crosses. Additionally, 17 mutants were eliminated from the array setup due to very slow growth. The final E-MAPs contained 146'412 genetic interactions and interaction profile correlations. In addition, some genetic interactions were eliminated by the software for different reasons (gene proximity, missing colonies, etc.) resulting in the final MSP- and MSP/C-E-MAPs containing the 142,108 and 142'114 validated genetic interactions listed in S2A Table. After cleaning, the final array set contained 543 strains and the final query set 504 strains (S6 Table). Thus, the final MSP-E-MAP scores were calculated from validated 504 reciprocal and 39 one way crosses (S2 Table). Strains were always eliminated from both datasets obtained without and with Cerulenin. Removal of noisy strains indeed increased the correlation between reciprocal crosses (S1A Fig - S1D Fig) and removed almost many genetic interactions located along the x- and y-axis in S1A Fig, to yield the validated interactions of non-noisy strains shown in S1B Fig. Some non-noisy strains still gave different values in reciprocal crosses, mainly the ones showing positive S scores on Cerulenin (Fig 1E, S1D Fig); for such strains the final S scores listed in S2A, S4 and S3 Tables and reported as heat map in S8 Fig always contained the averaged S scores comprising the values of the two (uneven) reciprocal crosses. The final dataset is reported by 543 x 543 heat maps and matrices (S8 Fig; S4 and S3 Table). Of the [(543 x 543) - 543]/2 =147'153 theoretical combinations of two different genes in such a matrix, [(39 x 39) - 39]/2 = 741 were not tested, and 504 x 39 = 19'656 were tested only in one way, whereas [(504 x 504) - 504]/2 = 126'756 were tested by reciprocal crosses. 84 randomly chosen queries were crossed with the array in a repeat experiment, generating again [(84 x 84) - 84]/2 = 3486 reciprocal crosses, and 38556 one directional crosses. These repeat data were also integrated into the final data reported in S2, S4, and S3 Tables and S8 Fig, which thus show the average of all data obtained for each given gene combination. Significant genetic interactions were generated by 528 of the 543 genes of the E-MAP set (S2B Table).

*Hierarchical clustering*

For S8 Fig, the matrix containing all correlations (S4B Table) was subjected to hierarchical clustering, thus generating a “correlation of correlations matrix”, i.e. juxtaposing genes having the same profile of correlations and resulting in the matrix of S3B Table. The corresponding heat map is in S8B. Substituting genetic interaction scores into the same hierarchical clustering canvas using the matrix of S3A Table generated S8A Fig. When the matrix of genetic interactions (S4A Table) was used as a base of the hierarchical clustering process, i.e. when data were clustered based on the genetic interaction profiles, we obtained a very similar heat map, containing all the clusters and boxes of S8 Fig, although genes were in slightly different order. For the visualization of the data in heat maps, data from the final E-MAP toolbox file were first clustered using the Cluster 3.0 program then visualized using the Java Treeview program. Both of these software can be downloaded at <http://bonsai.hgc.jp/~mdehoon/software/cluster/software.htm#ctv>.

*Microsome preparation*

Cells grown during 16 h to an OD_600_ of 1 - 2 in YPD + 1.4 M sorbitol in the absence or presence of 10 µg/ml Doxy, were centrifuged and resuspended in zymolyase buffer (10 mM NaN_3_, 1.4 M sorbitol, 50 mM potassium phosphate pH 7.5, 40 mM β-mercaptoethanol, 1 mg ml^-1^ zymolyase 20T) followed by an incubation of 1 h at 30°C without shaking. Spheroplasts were collected by centrifugation at 800 x g for 5 min at 4 °C and washed with one volume of zymolyase buffer lacking β-mercaptoethanol and zymolyase. Spheroplasts were lysed in lysis buffer (1.4 M sorbitol, 3 mM MgCl_2_, 50 mM potassium phosphate pH 7.5, 5 μg ml^-1^ pepstatin A and 1 x EDTA-free Roche protease inhibitor cocktail) by forcing them 20 times through a 0.4 mm gauge needle. After centrifugation at 1000 x g for 5 min at 4 °C, the supernatant was centrifuged at 16000 x g for 60 min at 4 °C to pellet the microsomes. The supernatant was discarded and microsomes were resuspended in small volumes of lysis buffer lacking protease inhibitors.

*Metabolic radiolabeling of cells and microsomes*

For the labeling of lipids with [^3^H]-C16:0 (Fig 4A - 4C), 10 OD_600_ of WT or 1∆2∆3ty∆ cells grown with or without 10 µg/ml Doxy for the indicated times in YPD or YPD + 1.4 M sorbitol, were centrifuged and resuspended in 2 ml SC medium supplemented with 10 µg/ml Cerulenin and preincubated for 20 minutes at 30°C. Twenty µCi (≅ 0.3 µM) [^3^H]-C16:0 were then added and the cultures incubated at 30°C for the indicated time periods. The lipids of all assayed cells were extracted, treated with NaOH if indicated, spotted and separated by TLC with solvent 1. Labeling of lipids with [^3^H]-*myo*-inositol (Fig 4D) was done by growing cells in inositol-free SC medium containing 1.4 M sorbitol and supplemented or not with 10 µg/ml Doxycycline for 16 hours. Aliquots of 10 OD_600_ units of cells were collected, centrifuged and resuspended in 1 ml fresh medium of the same kind, 40 μCi of [^3^H]-*myo*-inositol were added (t = 0) and the cells were put back to 30°C without cover for good aeration. Fresh medium (0.2 ml) from a 5x concentrated stock was added at t = 40 and 80 min. Labeling was stopped after 2 h. Lipids of 10 OD_600_ units of cells were extracted, spotted and separated by TLC with solvent 2.

For labeling of lipids in microsomes (Fig 5), 50 µg of microsomal protein were incubated at room temperature for the indicated times in 1.4 M sorbitol, 3 mM MgCl_2_, 50 mM potassium phosphate pH 7.5 with 6.6 µM (0.1 µCi) [^14^C]-G3P and the indicated concentrations of C16:0-CoA and Triton X-100 in a final volume of 100 µl. Lipids were extracted and separated by TLC with solvent 1. Labeling of GPI anchor lipids in cells (Fig 10C) and their isolation was done as previously described in [10] except that after washing, the elution of anchor peptides from octyl-Sepharose was done directly with 50% propanol and that twice the number of cells and double amounts of radioactivity were used to label *per1∆, gup1∆* and *gup1∆ cwh43∆* mutants and that therefore, for these strains, doubled amounts of lipids were spotted on the TLC shown in Fig 10C. Anchor lipids were resolved on TLC in solvent 3. The microsomal labeling of GPI lipids (Fig 8), was done as previously described in [11] but with the following modifications: 100 µg of microsomal protein in a final volume of 100 µl were incubated at room temperature for the indicated times in buffer (1.4 M sorbitol, 50 mM potassium phosphate pH 7.5, 3 mM MgCl_2_, 0.5 mM MnCl_2_, 1 mM EGTA) supplemented with 1 mM Coenzyme A, 1 mM ATP and 21 µg/ml Tunicamycin except in one control where ATP and CoA were omitted and 1 mM Glycerol-3-phosphate was added.

*Metabolic radiolabeling of permeabilized cells*

Cells were grown for 16 h in YPD + 1.4 M sorbitol with or without 10 µg/ml Doxy and washed with buffer (50 mM potassium phosphate pH 7.5, 1.4 M sorbitol, 10 mM NaN_3_, 3 mM MgCl_2_). Cells were resuspended at an OD_600_ of 10 in the same buffer and aliquots of 1 ml were supplemented with different concentrations of Digitonin as indicated. Then the 1 ml aliquots were preincubated during 30 minutes at room temperature to allow the permeabilization of the plasma membrane. The acyltransferase activities of cells were assayed at room temperature by adding either C16:0-CoA (10 µM final) and [^14^C]-G3P (0.5 µCi, 10 µM final)(Fig 6 and S5 Fig), or [^3^H]-C16:0-CoA (1 µCi, 10 µM final) and other ingredients as indicated (Fig 7)*.* Lipids were extracted and separated by TLC with solvent 1.

*Lipid extraction from cells and microsomes*

Lipids were extracted from cell pellets by first mechanically disrupting the cells with glass beads in chloroform-methanol (2:1), the lysate was then centrifuged and the pellet further extracted with 95% ethanol-water-diethyl ether-pyridine-4.2 N ammonium hydroxide (15:15:5:1:0.18). The supernatants containing the lipids were pooled, dried and desalted by butanol-water partitioning as described [10]. When microsomes were assayed, butanol was used to stop the reactions and extract the lipids. To extract radiolabeled GPI lipid intermediates from microsomes (Fig 8), 667 µl chloroform-methanol (1:1) was added yielding a final ratio of chloroform-methanol-water of 10:10:3 and the tubes were vigorously vortexed. The tubes were centrifuged at 10’000 g for 5 minutes and the supernatant transferred to a separate tube. The pellet was extracted once more with chloroform-methanol-water (10:10:3) and the pooled supernatants were dried. The dried lipids were desalted by butanol–water partitioning as described by [10], dried again, resuspended and separated on TLC using solvent 4.

*Lipid analysis*

Lipids were analyzed by TLC on silica gel 60 glass plates. The solvents used in this study were solvent 1 (chloroform-methanol-0.25% KCl, 55:45:5), solvent 2 (chloroform-methanol-2N ammonia, 9:7:2), solvent 3 (chloroform–methanol–0.25% KCl, 55:45:10) or solvent 4 (chloroform–methanol–water, 10:10:3). For Fig 7, PI was distinguished from PA by scraping the silica from TLC plates (except for origin), extracting the silica with solvent 4 and treating the dried extract in NEBuffer 3™ (New England Biolabs) supplemented with 0.1% deoxycholate with or without 50 U alkaline phosphatase (AP) at 37°C for 2 h. The lipids were extracted twice from the aqueous solution by adding chloroform-methanol (2:1), the organic phases were pooled and dried, resuspended in solvent 4, spotted and separated again by TLC with solvent 1. For mild base treatment, dried lipids were resuspended in 200 μl chloroform–methanol– water (10:10:3), 40 μl of 0.6 M NaOH in methanol was added, and samples were incubated at 37°C for 1 h. Hydrolysis was stopped with 40 μl of 0.8 M acetic acid, and the lipids were dried. Control samples followed the same procedure, but NaOH and acetic acid were added together at the end of the incubation. Dried lipids were desalted by butanol–water partitioning as described by [10].

**Supplemental Figure legends**

**S1 Fig. Processing of raw E-MAP data.** (A - D) S scores of double mutants observed on [query A x array B] plates vs. S scores of same double mutants observed on [query B x array A] plates are plotted. For this, for each gene combination, the software averages the size of the two colonies on the [query A x array B] and on the [query B x array A] plate separately and thereby generates a so-called unaveraged score for each [9]. Thus, the x- and y-coordinates of each dot indicate these unaveraged S scores obtained in the two reciprocal crosses ([*aaaA::ura3MX* x *bbbB::kanMX*] and [*bbbB::ura3MX* x *aaaA::kanMX*]). (Crosses done only in one direction are not plotted). (A, B) S scores of MSP-E-MAP before (A) and after (B) removal of noisy strains. (C, D) S scores in MSP/C-E-MAP before (C) and after (D) removal of noisy strains. Panels (B) and (D) correspond to Fig 1D and 1E and are shown here once more to demonstrate that elimination of noisy strains increases the correlation between unaveraged S scores obtained in reciprocal crosses. (E*,* F) the similarity between reciprocal crosses can also be seen when calculating medians of the second unaveraged S scores as a function of first unaveraged score using the software of [9,12]. The plot was calculated after having removed noisy strains. (E) MSP-E-MAP; (F) MSP/C-E-MAP. The not quite proportional lines in (E) and (F) are very similar to the ones obtained in the ESP-E-MAP, i.e. Figure S1 of [12] and Fig 3b of [9]. (G, H) with the data of Fig 2, the ratio of the numbers of gene pairs giving significantly positive over significantly negative S scores was calculated as a function of the correlation for the MSP-E-MAP (G) and the MSP/C-E-MAP (H) using a window of 30 values.

**S2 Fig. Reproducibility of correlations of the MSP-E-MAP and MSP/C-E-MAP.** (A, B) a subset of 84 queries were crossed once more with the 629 genes of the array without (A) or with Cerulenin (B) by different persons (authors Christine Vionnet and Carole Roubaty ) and the interaction profile correlations compared to those obtained in the first MSP- and MSP/C-E-MAP (carried out by author Hector M. Vazquez) as described in Fig 1F, 1G legend. The trend lines and the corresponding Pearson correlation values of the two replications are for all the correlation coefficients (black) or only statistically significant correlations (blue). The dashed line represents a correlation of R = 1*.*

**S3 Fig. Division times of single or combined *flc* mutants.** (A) WT, single or multiple *flc* mutants (labeled as in Fig 3A) were inoculated in 200 µl YPD containing or not 0.1, 1 or 10 µg/ml Doxycycline and their growth rates were measured using an automated Bioscreen C MBR reader. Three independent strains were tested for each genotype. (B) mean division times were calculated from the exponential growth phase of curves of panel A.

**S4 Fig. Permeabilization of cells with Digitonin and detection of cytosolic thiol groups with DTNB.** WT or 1∆2∆3ty∆ cells grown for 16 h in YPD + 1.4 M sorbitol with (A, B) or without (C) 10 µg/ml Doxy were centrifuged and resuspended in buffer (50 mM potassium phosphate pH 7.5, 1.4 M sorbitol, 10 mM NaN_3_, 3 mM MgCl_2_) at an OD_600_ of 20. Then, 500 µl of the same buffer supplemented with 2 x the desired concentration of Digitonin plus 2 mM DTNB were mixed with 500 µl of the cell suspension and the absorbance was continuously measured at 412 nm at room temperature. The curves in the charts are the average of two independent biological replicates.

*Interpretation:* Intact cells are resuspended in the same buffer and at the same concentration as used for labeling permeabilized cells (Fig 6, S5 Fig), but with DTNB. The increase of absorbance in presence of cells (only cells, no detergent) is only slightly faster than the spontaneous hydrolysis of DTNB observed in the samples without cells (panels A, B), suggesting that the cells remain intact and that, as previously described, the DTNB cannot penetrate the plasma membrane [13,14]. When adding 0.001% or higher concentrations of Digitonin, the plasma membranes of WT and 1∆2∆3ty∆ cells begin to lyse and DTNB starts to react with the sulfhydryl groups, mainly of proteins which remain in the cytosol [13,14]*.* The speed of this reaction increases with the concentration of Digitonin. 1∆2∆3ty∆ cells grown in Doxy are rather more resistant to plasma membrane solubilization than WT cells (panel B vs. A), and also slightly more resistant than 1∆2∆3ty∆ cells grown without Doxy (panel B vs. C). 1∆2∆3ty∆ cells seem to retain a full redox potential as the reaction reaches plateau at similar values as in WT and since at high concentrations of Digitonin (0.02%) the reaction rate is the same for 1∆2∆3ty∆ cells and WT.

**S5 Fig. 1∆2∆3ty∆ mutants have normal GPAT and AGPAT activity when not incubated with Doxy.** (A, B) acyltransferase activity of WT and 1∆2∆3ty∆ cells was assayed with 10 µM C16:0-CoA and 10 µM [^14^C]-G3P after preincubation in 0.001% (A) or 0.005% (B) Digitonin.

**S6 Fig. Comparison of growth rates of *elo3∆, cst26∆* and *elo3∆ cst26∆* cells.** (A, B) exponentially growing cells were inoculated at an OD_600_ of 0.1 and grown in YPD at 30°C. OD_600_ values were measured at regular intervals. Four different *elo3∆ cst26∆* strains derived from different spores generated by tetrad dissection were tested, two in (A), two in (B).

**S7 Fig. Growth defects of mutants in the right arm of Chromosome II combined with *chs1∆*.** (A) growth of double mutants giving strong negative S scores (Fig 11A) was assayed in liquid culture using the Bioscreen C MBR reader allowing to calculate doubling times. The same medium as in the last selection step of the screen was used. The only double mutant having a significant difference with regard to corresponding single mutants is the unrelated *gup1∆ cwh43∆* strain*,* which has an S score of - 9.9 and was added here as a positive control. (B) double mutants containing *chs1∆* plus a deletion of some gene on the right arm of Chr. II together with corresponding parental single mutants were plated in four fold serial dilutions on the same medium as used in the last selection step of the MSP-E-MAP.

**S8 Fig: Heat maps and main clusters of the MSP-E-MAP.** Hierarchical clustering generated heat maps showing the genetic interaction S scores (A) and profile correlations (B) of the MSP-E-MAP. The main clusters are highlighted by circled numbers. Zones of frequent interactions or correlations outside the diagonal are either boxed or annotated at the right side of panel A by constituent gene names. In (A), gray dots indicate "no value" because gene combinations were not tested or values were eliminated for one of several reasons (see *Data clean up*). (C) enlarged views of the clusters placed into boxes in (A) and (B). Genes making up the clusters are indicated at the left in color code according to the functional categories attributed in S1 Table. The string of color codes is repeated at the top for easy reference. The interaction of the "microcluster" of *ELO3/SAC1* with genes of cluster 1 is shown to the right. Further enlargements of colored regions outside the diagonal showing a high density of interactions and/or correlations between clusters are shown in S9 Fig.

**S9 Fig.** **Enlargement of regions in heat maps of S8A and S8B Fig showing frequent interactions or correlations between genes belonging to two different clusters.** To the left, the clusters shown in S8C Fig are shown once more, spelling out the gene names making up each cluster in color code mode to indicate their functional category (S1 Table). More to the right follow cluster intersections. In all cluster intersections the same zone is shown once from the correlation map (S8B Fig) and once from the interaction map (S8A Fig), side by side. For instance, in the first row, the enlargements of the heat maps show interactions and correlations of genes of cluster 1 with genes of clusters 2, then 3, then 4, then 5 and finally, to the right, cluster 6. On top of each cluster intersection, the color codes of the intersecting cluster are repeated twice, once for the interaction map, once for the correlation map. Some zones with frequent interactions outside the cluster intersections are also shown and the names of the genes in those zones are indicated above the top row but are the same for all zones below. The figure highlights for instance the negative interactions and correlations between clusters 3 and 5 or the positive interactions between clusters 5 and 6, the positive interactions of *ilm1∆* with cluster 6 and intriguing negative interactions of *sur1∆* (=*csg1∆*) with cluster 5 mentioned also in the text.

**S10 Fig. Frequency of significant interactions and correlations within and amongst different functional classes of genes.** (A) as introduced in S1 Table, each of the 543 genes of the E-MAP set was allocated to one of 11 functional categories represented by the color code on the left and repeated at the top and at the bottom. The number of genes present in each category is indicated at the far left. Each square gives the number of significant negative (upper, olive) or significant positive (lower, orange) S scores (below diagonal) or correlations (above diagonal), as a percentage of the total number of gene combinations possible between the category indicated at the left end of row and the category at the bottom or top of the column. For columns, the functional category is only indicated by repeating the color code used for rows. If interactions were randomly distributed one would expect for negative and positive interactions values of 0.4 and 0.6 %, for negative and positive correlations of 0.4 and 0.6 %, respectively. (B) the same as in (A) but for the MSP/C-E-MAP. Here, only those percentages, which underwent a significant change (P value ≤ 0.05) in comparison with the MSP-E-MAP are shown. In all panels, the intensity of olive and orange coloring of values is proportional to the percentage.

**S11 Fig.** **Interdependence of the number of interactions and correlations generated by the MSP-E-MAP**. (A) from the matrix of interaction scores of the MSP-E-MAP (S4A Table), the number of significant positive and significant negative genetic interactions was counted for each one of the 543 genes of the E-MAP set using the COUNTIF function in EXCEL as detailed in S6A Table. Each gene is represented by a dot with x and y coordinates indicating the number of its significant positive and its significant negative interactions, respectively. The correlation coefficient R as well as the average number of positive and negative interactions is indicated. (B) is as (A), yet not the number of interactions, but rather the sum of the positive and of the negative S scores in all significant interactions was calculated for each gene as shown in S7B Table. (C) the number of positive correlations was plotted as a function of the number of negative interactions for each of the 543 genes. (D) is as (A), but the number of correlations rather than interactions was obtained from the 543 x 543 correlation matrix (S4B and S7A Table). (E) from the 543 x 543 matrix of interaction and correlation scores (S4A and S4B Table), we tabulated for each gene the number of significant negative and positive genetic interactions as well as of significant positive and negative correlations it had generated. Then, the 543 genes of the E-MAP set were placed into 4 classes depending on whether they showed no, only positive, only negative or both types of genetic interactions; independently they were categorized into 4 classes depending on the correlations they had generated. By combining the 4 interaction and 4 correlation classes, a matrix with 16 categories was generated and each gene of the 543 attributed to one of these categories. Only 120 genes generated everything, negative and positive interactions and negative and positive correlations. (F) the correlations between the numbers of positive and negative interactions and positive and negative correlations generated by the 543 genes in both the MSP- and the MSP/C-E-MAP were calculated (S7D Table) and grouped into a Table giving in each field the correlation between the categories at the left of the row and at the top of the column. The green color is proportional to the correlation coefficient.

**S12 Fig. Comparison of E-MAPs with or without Cerulenin.** (A, B) In (A) the sides of the grey box indicate the significance thresholds of S scores of double mutants in the MSP- and MSP/C-E-MAPs as used in Fig 2. Similarly, in (B) the grey box gives the significance thresholds of profile correlations between any two genes according to Fig 2. Values that underwent a strong increase (P value ≤ 0.005 for interactions, ≤ 0.05 for correlations) in the MSP/C-E-MAP as compared to the MSP-E-MAP are in red, those that underwent a significant decrease are green. For instance, the red dots above the box in (B) designate gene pairs that were not correlated without Cerulenin, but positively correlated on Cerulenin. The trend lines and the corresponding Pearson correlation values for all dots are in black, those for dots outside the grey box in blue. The dashed line represents correlation of R = 1.

**S13 Fig. Titration of Cerulenin to determine its optimal concentration for the MSP/C-E-MAP.** The MSP-E-MAP array gene set was robotically replicated on plates containing 0, 0.4, 0.5 and 0.6 µg/ml Cerulenin and incubated for 24 h at 30°C before pictures were taken.

**Supplemental References (used in here and figure legends of supplemental figures)**

1. Breslow DK, Cameron DM, Collins SR, Schuldiner M, Stewart-Ornstein J, Newman HW, et al. A comprehensive strategy enabling high-resolution functional analysis of the yeast genome. Nat Meth. 2008;5: 711–718. doi:10.1038/nmeth.1234

2. Vazquez HM, Vionnet C, Roubaty C, Conzelmann A. Cdc1 removes the ethanolamine phosphate of the first mannose of GPI anchors and thereby facilitates the integration of GPI proteins into the yeast cell wall. Mol Biol Cell. American Society for Cell Biology; 2014;25: 3375–3388. doi:10.1091/mbc.E14-06-1033

3. Goldstein AL, Pan X, McCusker JH. Heterologous URA3MX cassettes for gene replacement in Saccharomyces cerevisiae. Yeast. 1999;15: 507–511. doi:10.1002/(SICI)1097-0061(199904)15:6<507::AID-YEA369>3.0.CO;2-P

4. Collins SR, Roguev A, Krogan NJ. Quantitative genetic interaction mapping using the E-MAP approach. Meth Enzymol. 2010;470: 205–231. doi:10.1016/S0076-6879(10)70009-4

5. Bavdek A, Vazquez HM, Conzelmann A. Enzyme-coupled assays for flip-flop of acyl-Coenzyme A in liposomes. Biochim Biophys Acta. 2015;1848: 2960–2966. doi:10.1016/j.bbamem.2015.08.020

6. Costanzo M, Baryshnikova A, Bellay J, Kim Y, Spear ED, Sevier CS, et al. The genetic landscape of a cell. Science. American Association for the Advancement of Science; 2010;327: 425–431. doi:10.1126/science.1180823

7. Baryshnikova A, Costanzo M, Dixon S, Vizeacoumar FJ, Myers CL, Andrews B, et al. Synthetic genetic array (SGA) analysis in Saccharomyces cerevisiae and Schizosaccharomyces pombe. Meth Enzymol. 2010;470: 145–179. doi:10.1016/S0076-6879(10)70007-0

8. Gietz RD, Schiestl RH. Microtiter plate transformation using the LiAc/SS carrier DNA/PEG method. Nat Protoc. 2007;2: 5–8. doi:10.1038/nprot.2007.16

9. Collins SR, Schuldiner M, Krogan NJ, Weissman JS. A strategy for extracting and analyzing large-scale quantitative epistatic interaction data. Genome Biol. 2006;7: R63. doi:10.1186/gb-2006-7-7-r63

10. Guillas I, Pfefferli M, Conzelmann A. Analysis of ceramides present in glycosylphosphatidylinositol anchored proteins of Saccharomyces cerevisiae. Meth Enzymol. 2000;312: 506–515.

11. Canivenc-Gansel E, Imhof I, Reggiori F, Burda P, Conzelmann A, Benachour A. GPI anchor biosynthesis in yeast: phosphoethanolamine is attached to the alpha1,4-linked mannose of the complete precursor glycophospholipid. Glycobiology. 1998;8: 761–770. doi:10.1093/glycob/8.8.761

12. Schuldiner M, Collins SR, Thompson NJ, Denic V, Bhamidipati A, Punna T, et al. Exploration of the Function and Organization of the Yeast Early Secretory Pathway through an Epistatic Miniarray Profile. Cell. 2005;123: 507–519. doi:10.1016/j.cell.2005.08.031

13. Battaglia G, Ryan AJ, Tomas S. Polymeric vesicle permeability: a facile chemical assay. Langmuir. 2006;22: 4910–4913. doi:10.1021/la060354p

14. Liu Q, Siloto RMP, Weselake RJ. Role of Cysteine Residues in Thiol Modification of Acyl-CoA:Diacylglycerol Acyltransferase 2 from Yeast. Biochemistry. 2010;49: 3237–3245. doi:10.1021/bi9020499
