# Supplementary figures and images for: Chemogenetic E-MAP in Saccharomyces cerevisiae for Identification of Membrane Transporters Operating Lipid Flip Flop
Source: PLoS Genet. 2016 Jul 27;12(7):e1006160. doi: 10.1371/journal.pgen.1006160 (PMC4962981; doi:10.1371/journal.pgen.1006160)

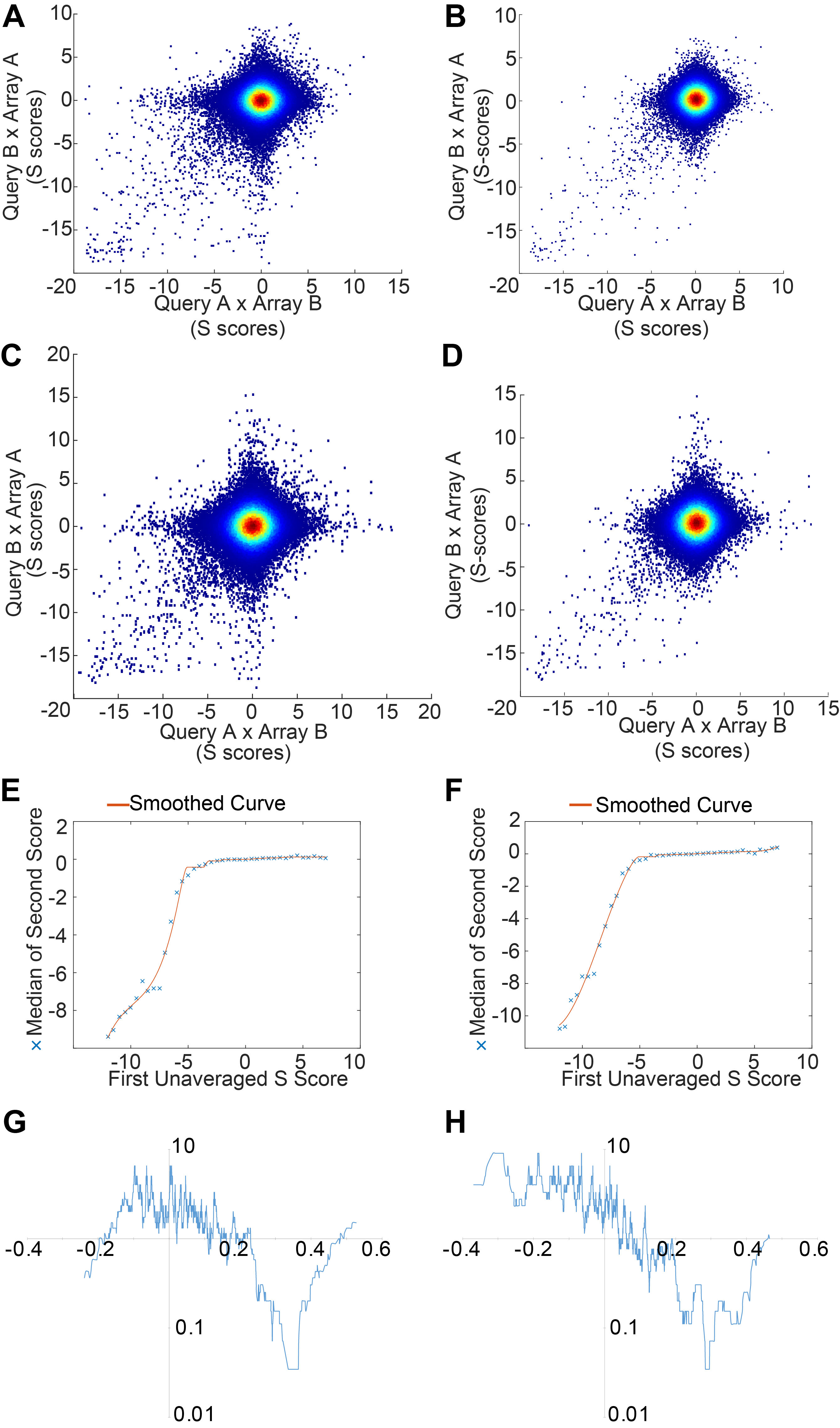

Supplement: S1 Fig — (TIF) [file pgen.1006160.s011.tif]

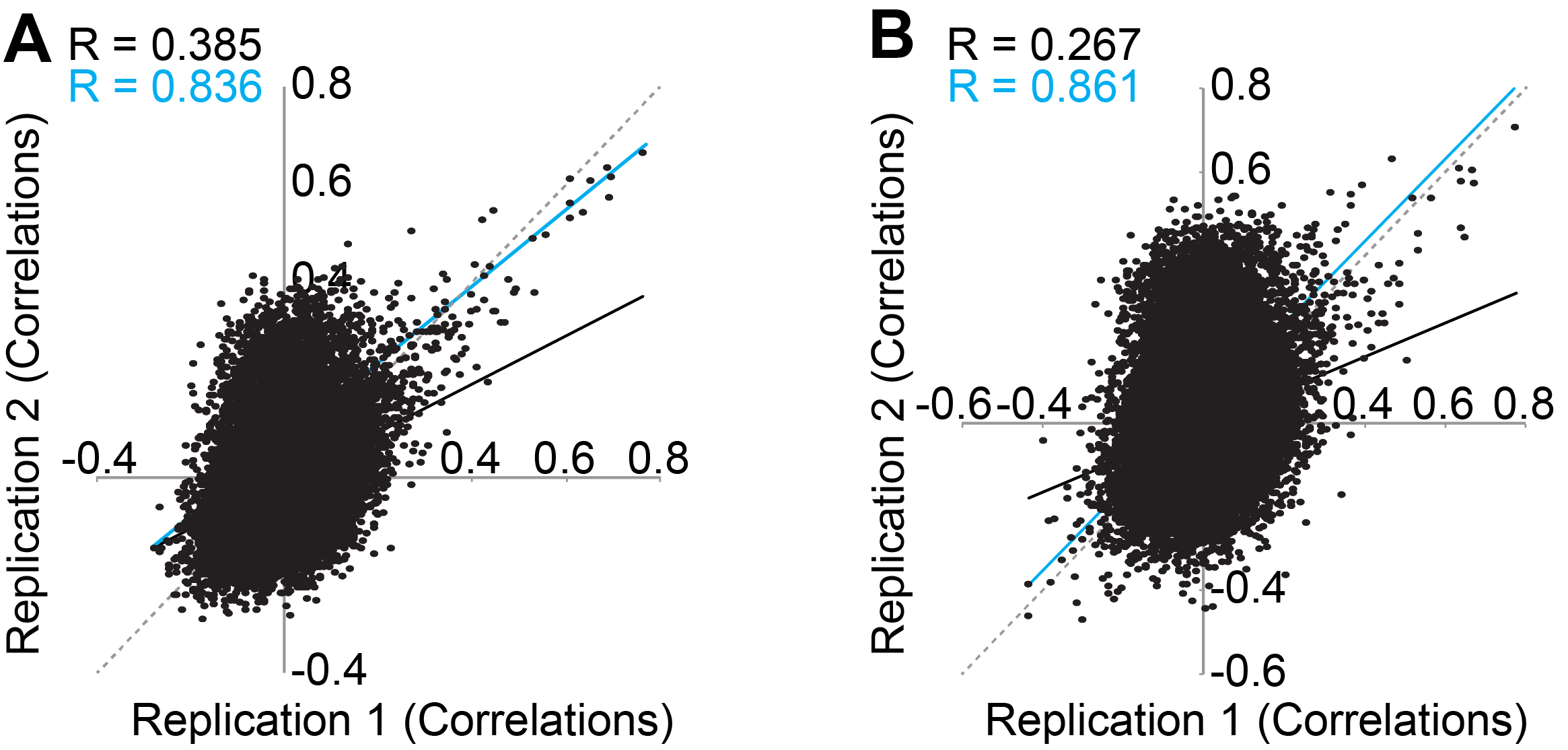

Supplement: S2 Fig — (TIF) [file pgen.1006160.s012.tif]

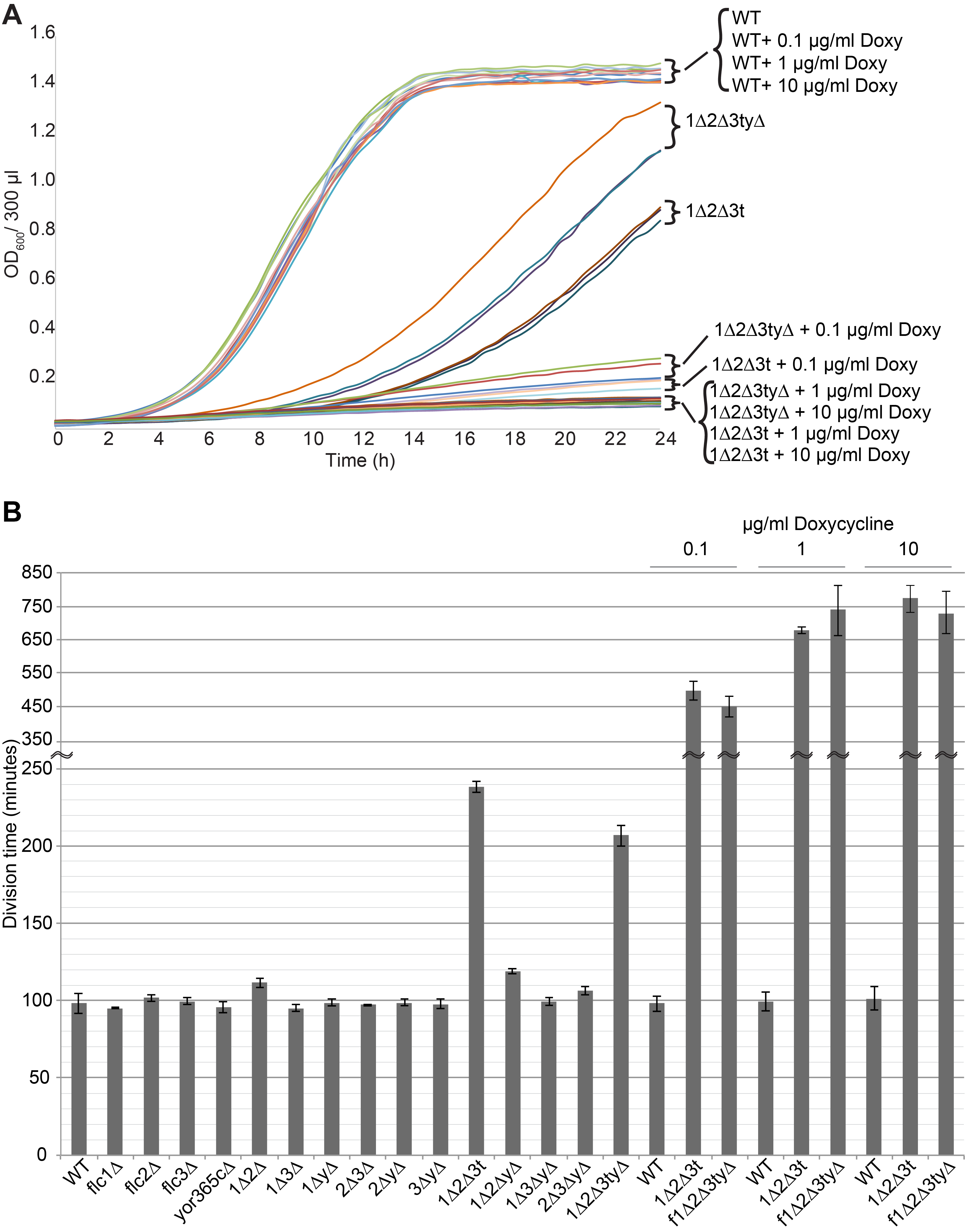

Supplement: S3 Fig — (TIF) [file pgen.1006160.s013.tif]

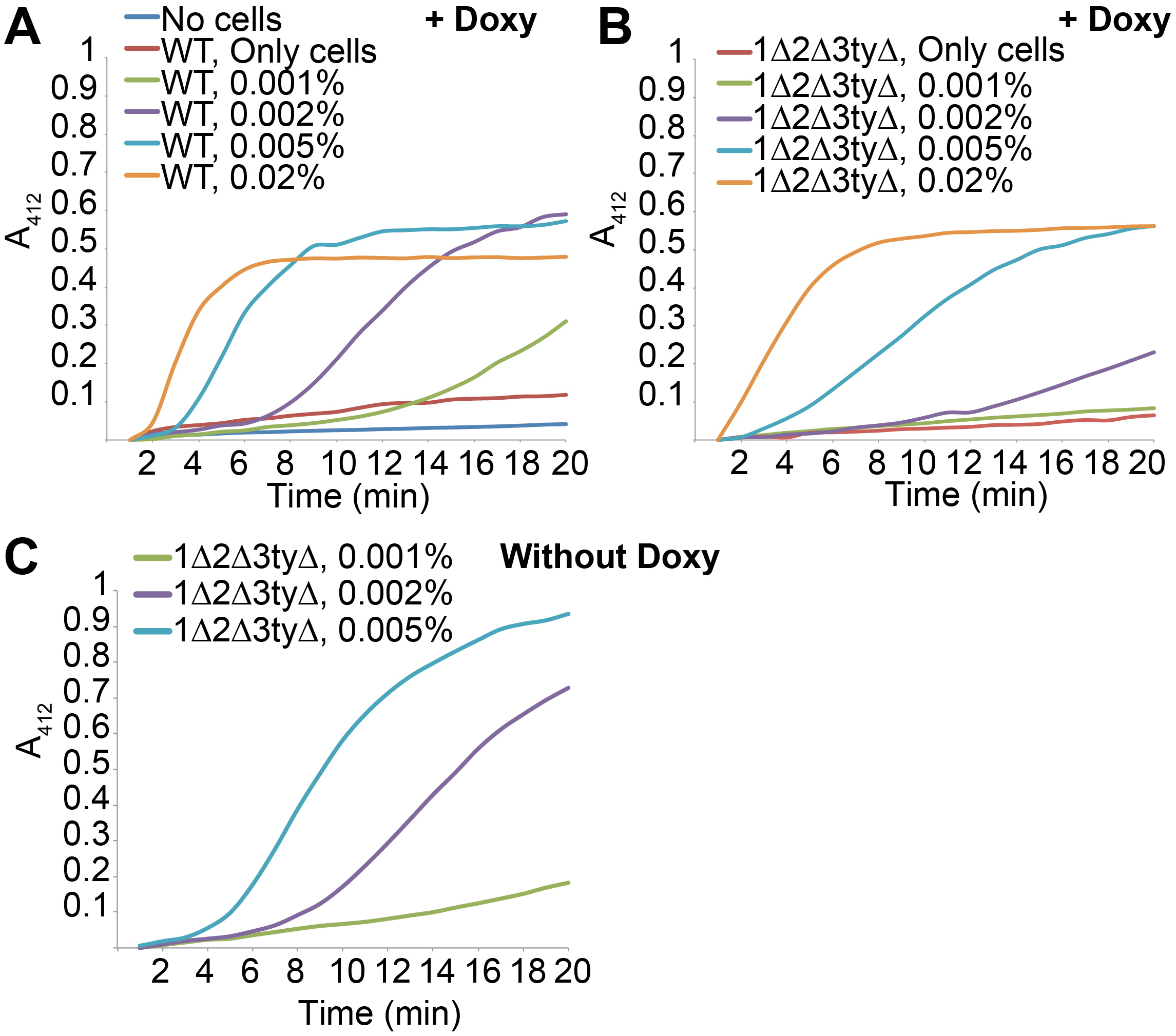

Supplement: S4 Fig — (TIF) [file pgen.1006160.s014.tif]

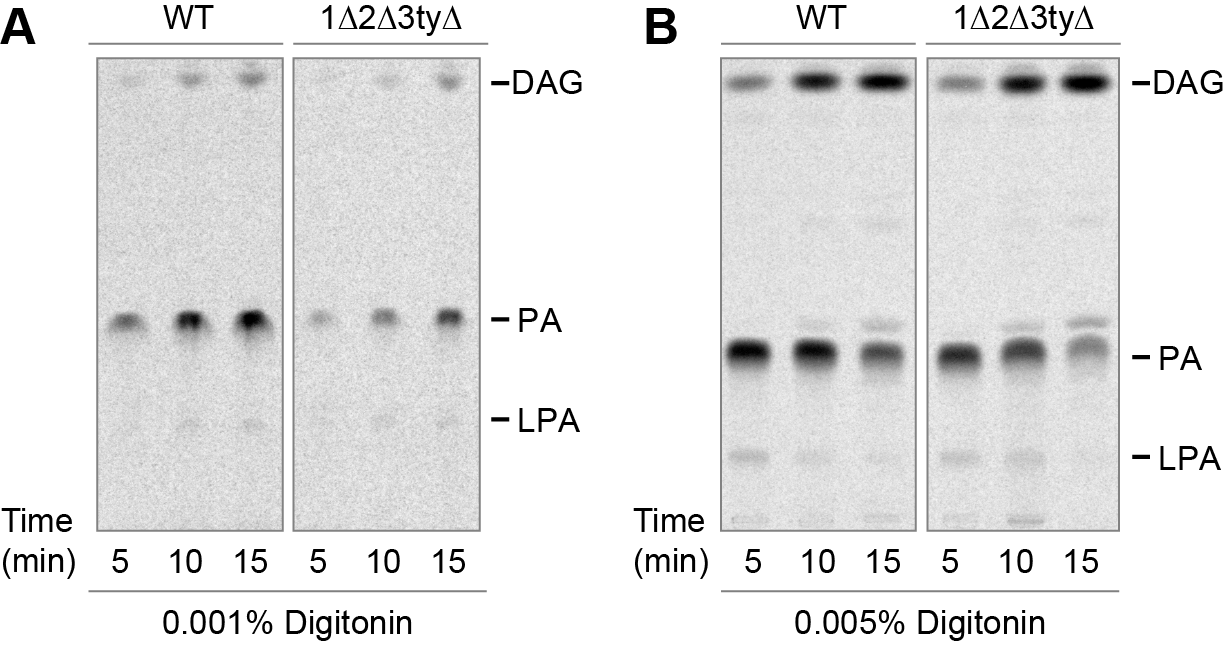

Supplement: S5 Fig — (TIF) [file pgen.1006160.s015.tif]

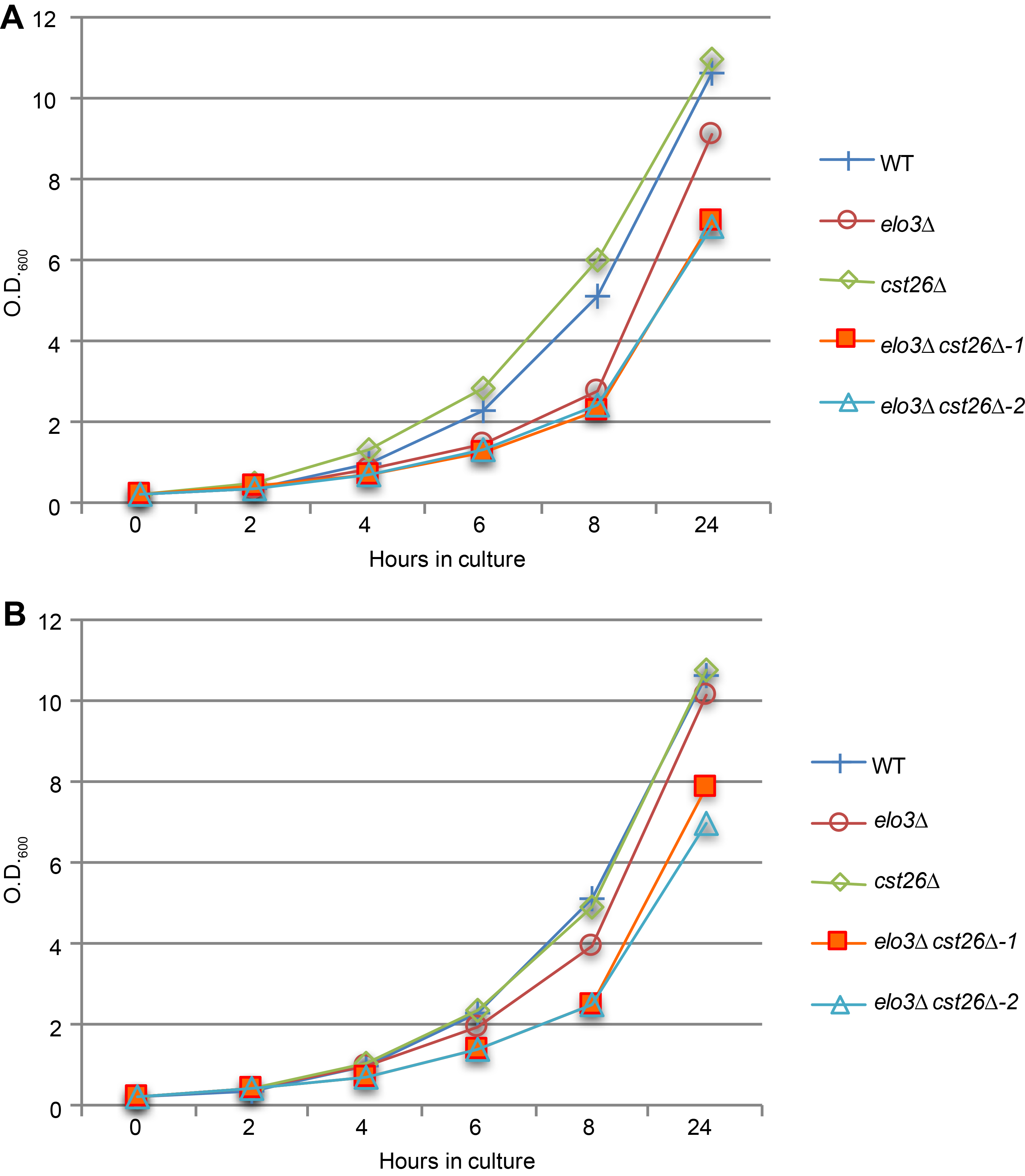

Supplement: S6 Fig — (TIF) [file pgen.1006160.s016.tif]

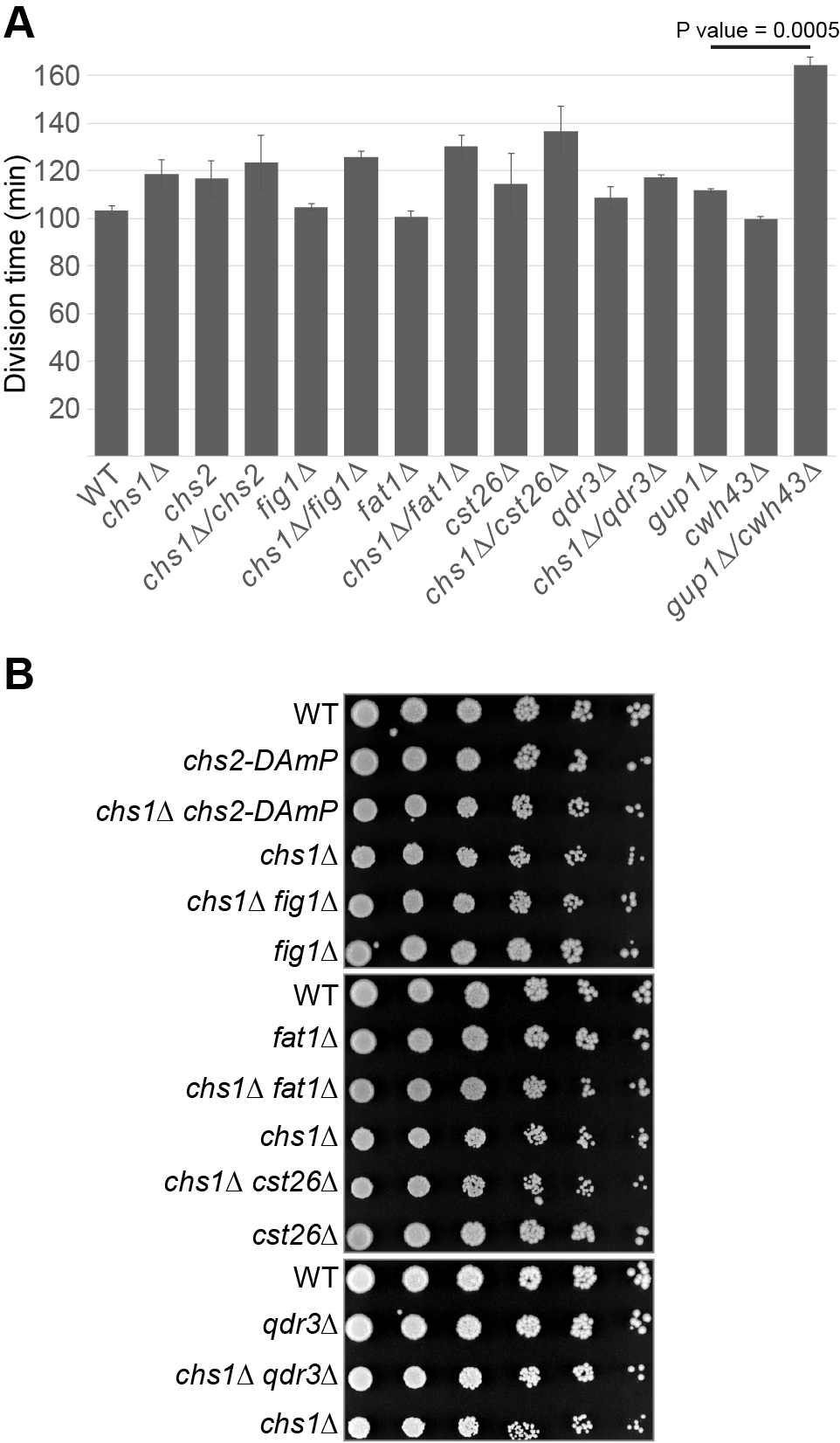

Supplement: S7 Fig — (TIF) [file pgen.1006160.s017.tif]

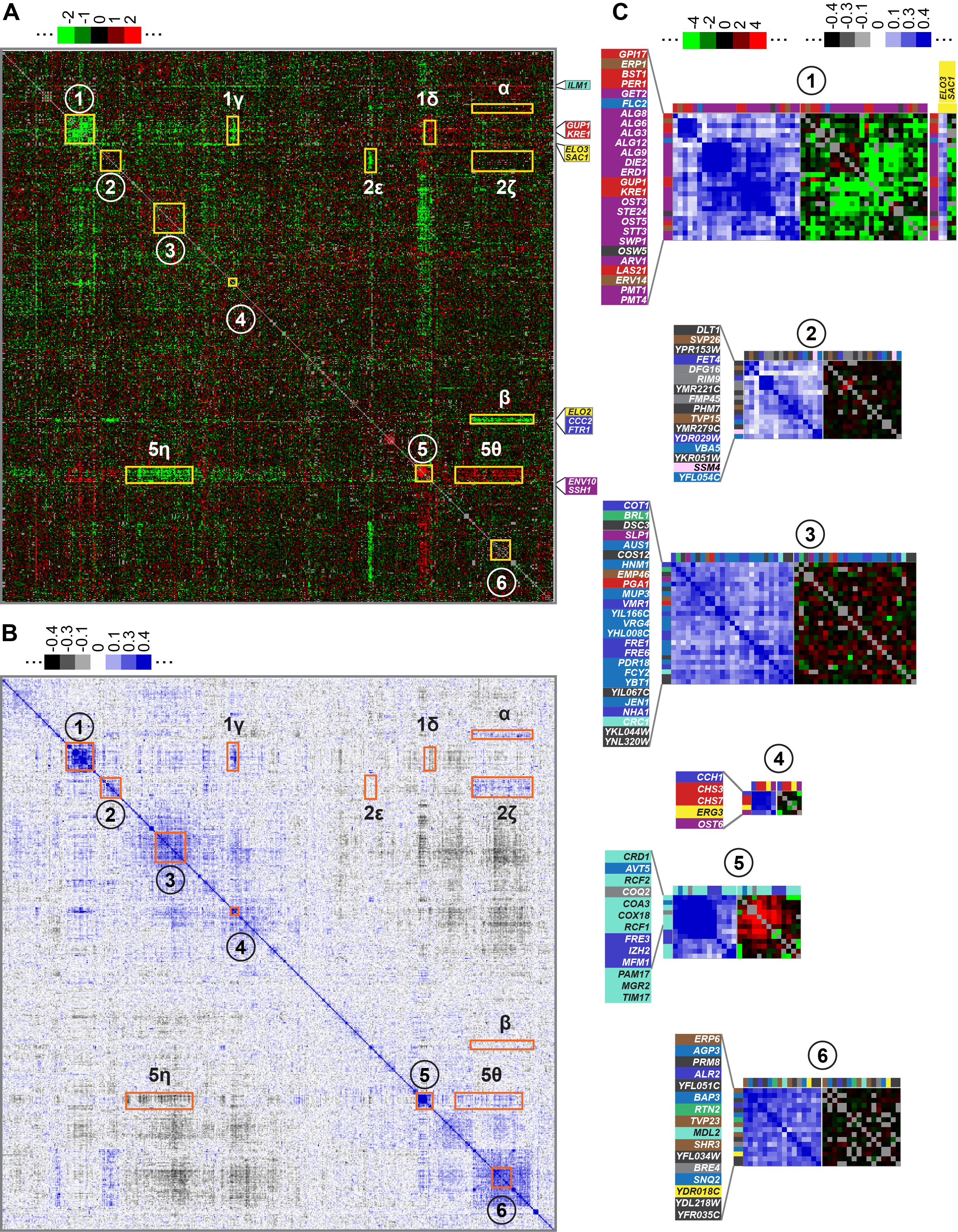

Supplement: S8 Fig — (TIF) [file pgen.1006160.s018.tif]

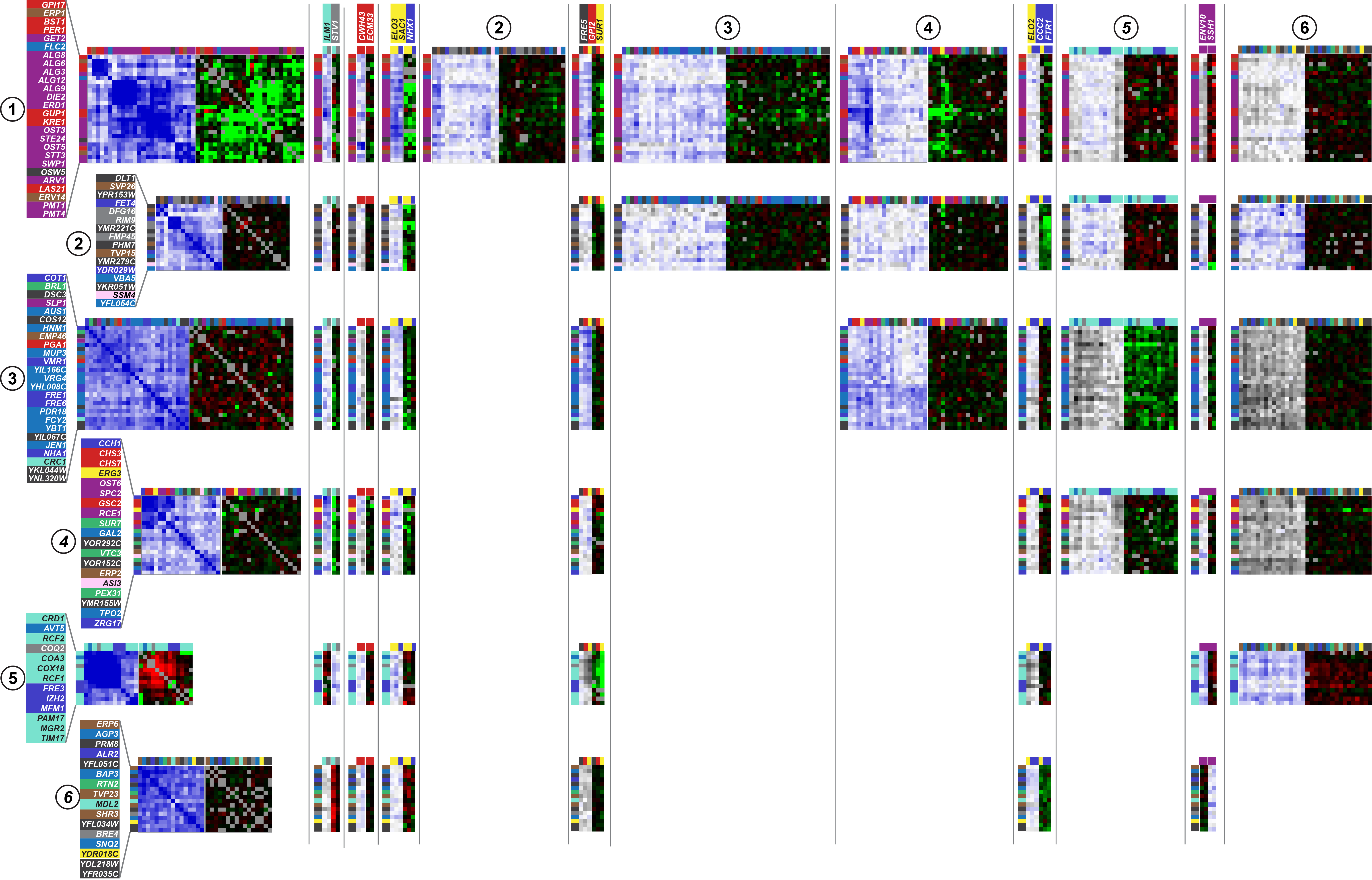

Supplement: S9 Fig — (TIF) [file pgen.1006160.s019.tif]

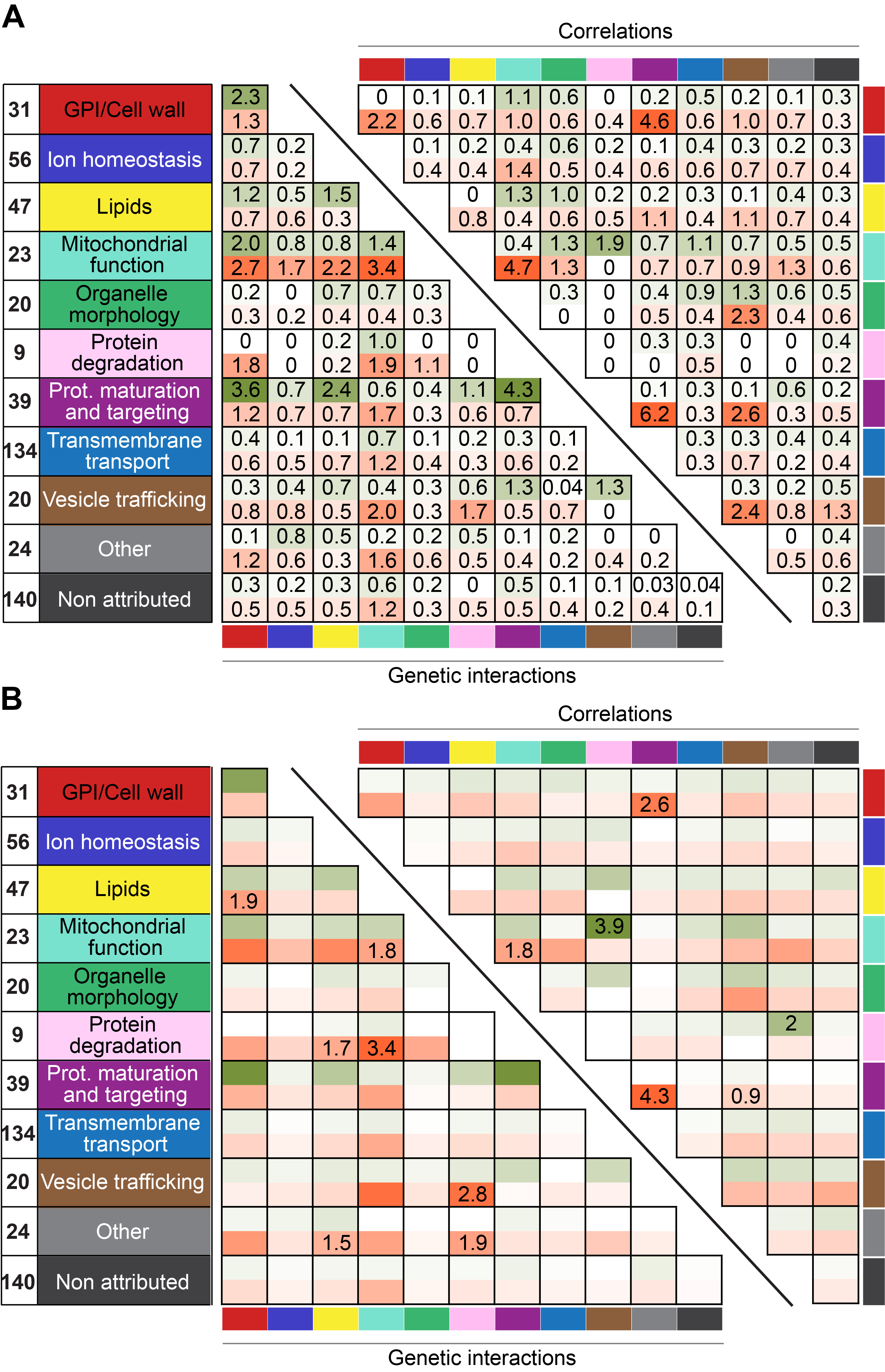

Supplement: S10 Fig — (TIF) [file pgen.1006160.s020.tif]

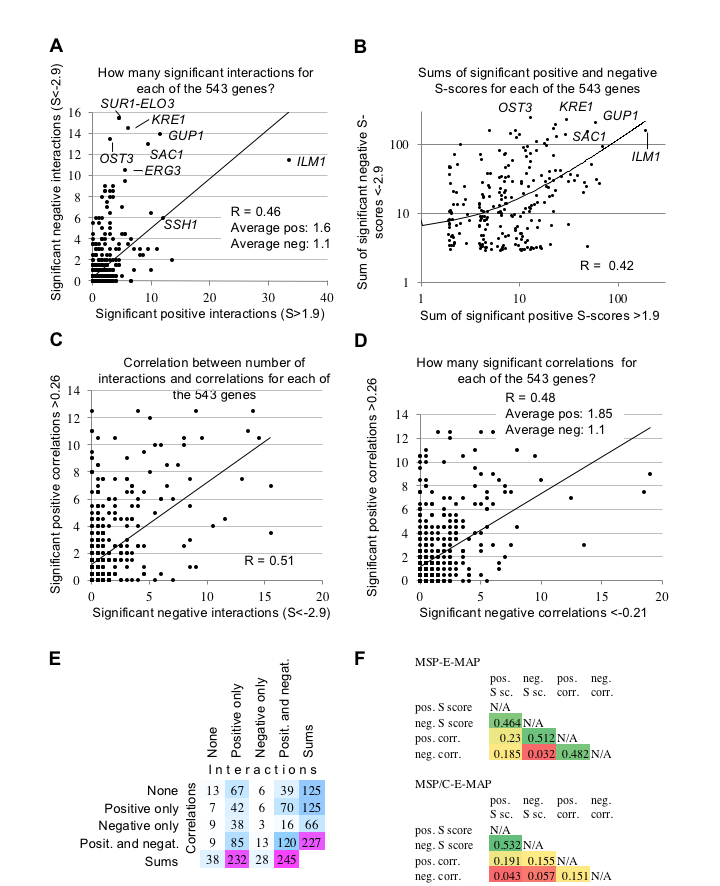

Supplement: S11 Fig — (TIF) [file pgen.1006160.s021.tif]

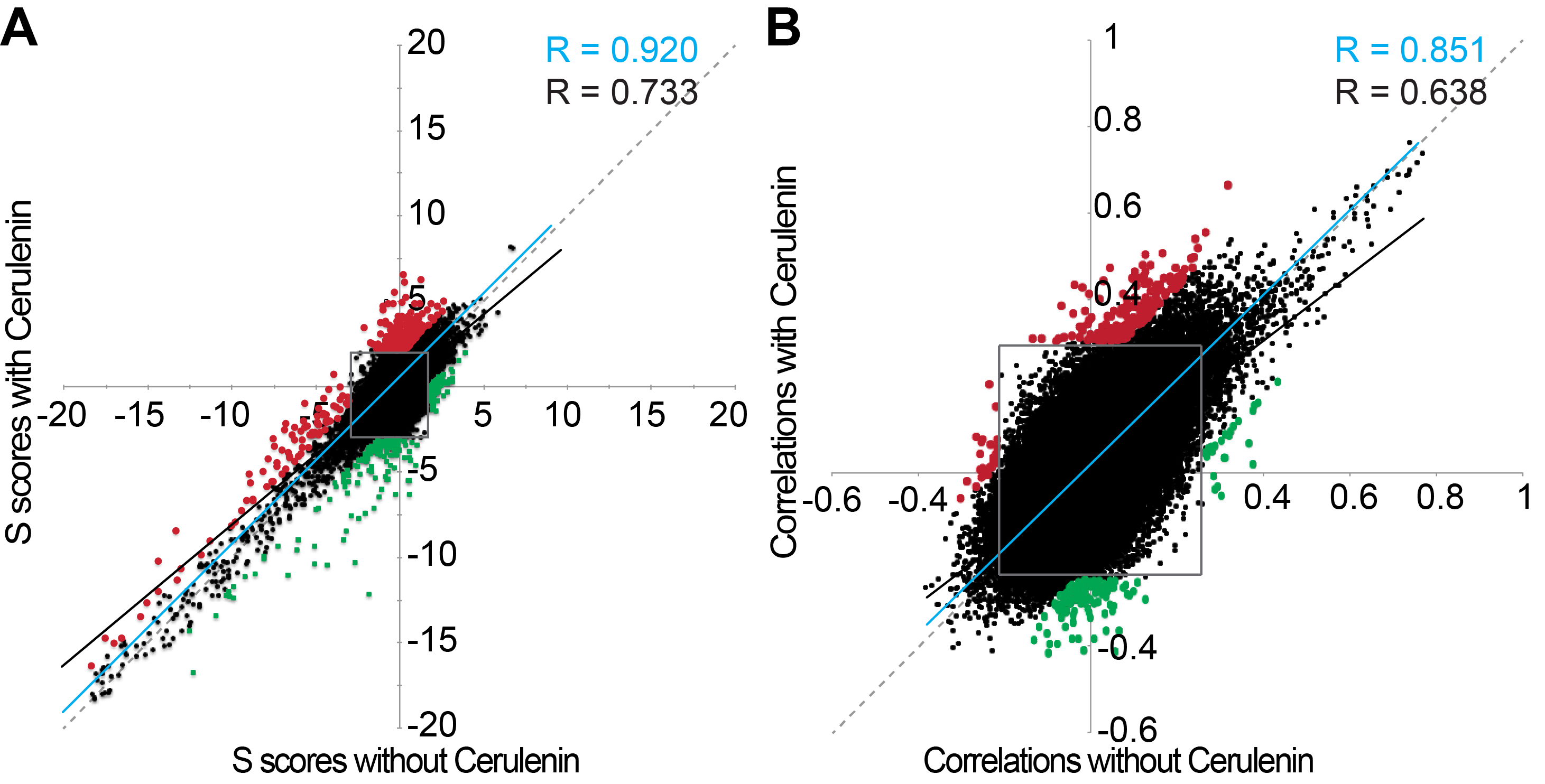

Supplement: S12 Fig — (TIF) [file pgen.1006160.s022.tif]

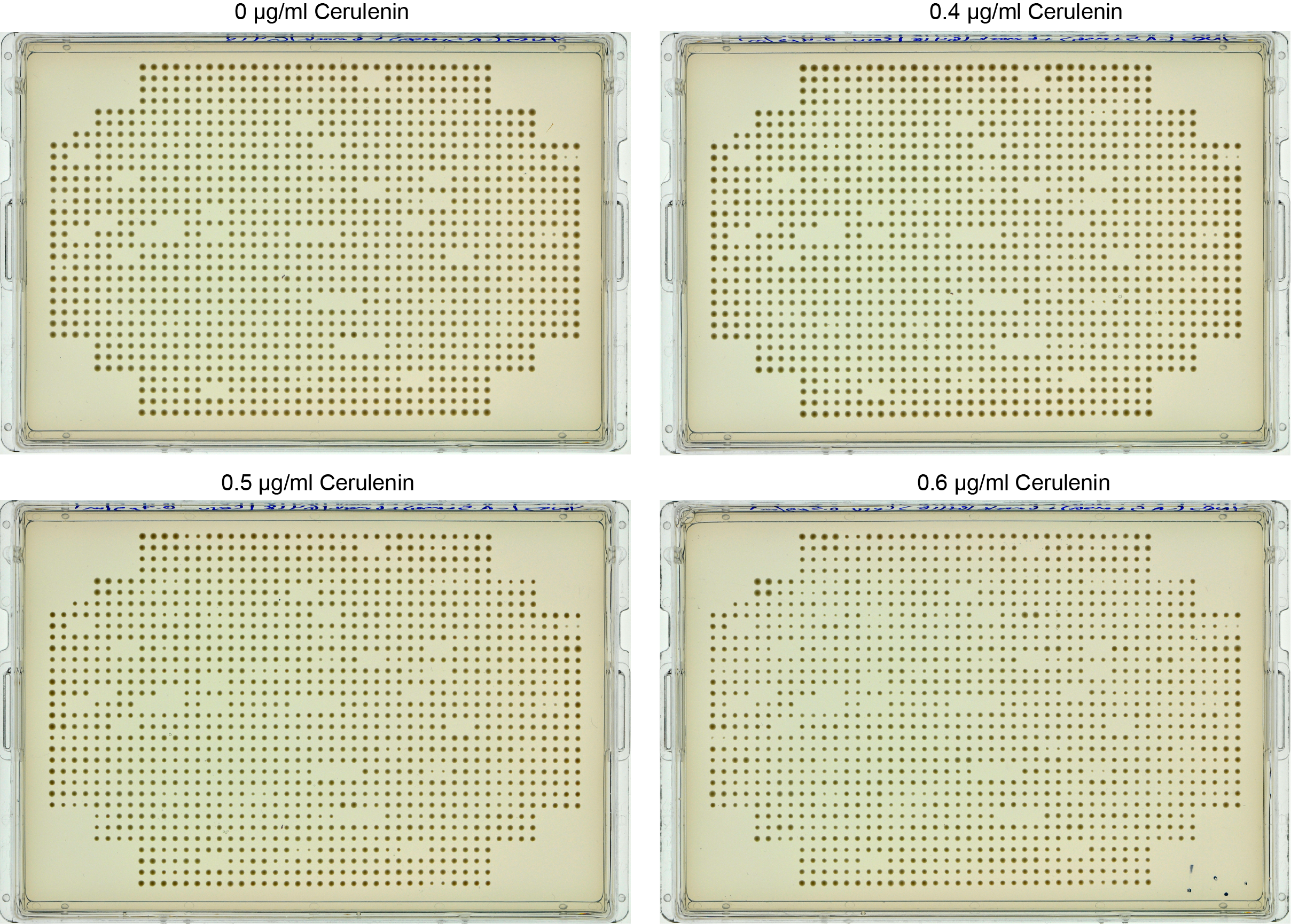

Supplement: S13 Fig — (TIF) [file pgen.1006160.s023.tif]
